# Paired growth of cultivated and halophytic wild rice under salt stress induces bacterial endophytes and gene expression responses

**DOI:** 10.3389/fpls.2023.1244743

**Published:** 2023-09-06

**Authors:** Anika Tasnim, Israt Jahan, Tomalika Azim, Dola Karmoker, Zeba I. Seraj

**Affiliations:** Plant Biotechnology Laboratory, Department of Biochemistry and Molecular Biology, University of Dhaka, Dhaka, Bangladesh

**Keywords:** mutualism, endophyte, salt tolerance, RNA-Seq, wild rice

## Abstract

**Introduction:**

Utilizing salt-affected marginal lands in coastal regions can help meet the growing demand for rice. We explored a nature-based solution involving wild halophytic rice (*O. coarctata, Oc*) and commercial rice BRRI Dhan 67 (*O. sativa, Os*) grown in close proximity to each other under salt stress.

**Methods:**

This was to investigate whether a paired planting strategy could help complement rice growth and yield under stress. We also investigated the gene expression and endophytic bacterial profiles of both *Os and Oc* in unpaired and paired conditions without and with salt.

**Results:**

Paired plants exhibited lower salt damage indicators such as smaller reduction in plant height, electrolyte leakage and chlorophyll loss, as well as higher K^+^/Na^+^ ratio under saline stress. Some of the 39 endophytic bacteria in the mutualism experiment were unique to *Oc* and transferred to *Os* when paired. Differentially expressed genes in leaves of paired *Os* versus unpaired *Os* were 1097 (994 up-regulated, 101 down-regulated) without salt and 893 (763 up-regulated, 130 down-regulated) under salt stress. The presence of *Oc* plants under salt stress influenced major biological processes in *Os*, including oxidative stress; chitinase activity; phenylalanine catabolic process and response to ABA. Protein binding and serine/threonine kinase activity were primarily affected in molecular function. The downregulated WRKY transcription factor 22 in paired conditions under salt stress played a role in the MAPK signaling pathway, reducing respiratory cell death. The upregulated auxin-responsive protein IAA18 gene, involved in hormone signaling and cell enlargement, was present only in paired plants.

**Discussion:**

Our findings therefore, offer insights into developing more effective cultivation strategies for sustainable rice production.

## 1 Introduction

Rice is a staple crop for over half the world’s population but it is very sensitive to salt stress. Many coastal and delta regions where rice is grown are affected by saltwater intrusion due to sea level rise which is causing vulnerability to the food security of resource poor farmers living in those regions and (Negrão et al., 2011). Developing rice varieties that can tolerate salt can help to increase crop yields in these areas and improve food security. Rice growth is the most sensitive to salinity stress at two developmental stages, early seedling and during reproduction but separate sets of genes may be involved at these phases (Walia et al., 2005). The physiological response to salt stress is myriad, with an immediate osmotic component evidenced by lower water uptake, lowering of cell expansion and growth retardation (Munns and Tester, 2008; Jadamba et al., 2020). Traditional rice cultivars or landraces which have evolved in the saline coastal regions have been found to be tolerant to salt stress. Such genotypes likely respond to this first phase of salt stress by controlling their stomatal apertures and producing compatible solutes (Horie et al., 2012). Plants subsequently experience ionic stress due to the eventual accumulation of Na^+^ ions. Tolerant genotypes are observed to maintain lower shoot Na^+^ concentrations (Rahman et al., 2016), due to sodium exclusion, sequestration of the salt into older leaves as well as roots and compartmentalization of Na^+^ into vacuoles and out of cells (Maathuis et al., 2014). There is also upregulation of the antioxidant machinery and other necessary genes such as, transcription factors (TF) and enzymes responsible for producing salt tolerance (He et al., 2020). Some tolerant genotypes manifest retrograde signaling (Huang et al., 2020), G-protein signaling as well as change in membrane potential (Razzaque et al., 2019), shape alteration in mesophyll chloroplast to allow greater dissipation of light energy (Yamane et al., 2018), maintenance of low reactive oxygen species (Bhattacharjee, 2012) and induction of RNA chaperones (Ganie, 2020).

Naturally occurring tolerant landraces therefore provide some hope as a resource for genes and their products, which can defend against stress, but these have met with limited success in breeding programs in terms of the level of tolerance obtained as well as maintenance of high yields (Munns and Tester, 2008). This is largely due to the complexity of the salt tolerance traits, lack of targeting all the sensitive developmental stages, types of genes involved in defense and or number of tolerance strategies as well as the background genotype of the recipient. Another approach could be use of crops which can perform reasonably well under saline conditions, enabling the use of brackish water or diluted seawater for irrigation (Morton et al., 2019). In order to improve saline soils and nutrient usage, synergies akin to symbiosis among perennial plants, halophytes, herbaceous plants, fungi, and bacteria have been discovered. Such synergistic actions within an ecosystem can be another approach to manage the effects of soil salinity and improve productivity (Ashilenje et al., 2023).

The wild halophytic rice (*O. coarctata*, *Oc*) locally referred to as Uri Dhan is endemic in the estuaries connected to the sea in all the Bangladeshi coastal shores. This distant relative of *O. sativa*, is the only known halophyte that can set rice-like grains, while growing naturally in extremely salty conditions ranging from 20 to 40 dS/m (Sengupta and Majumder, 2009; Garg et al., 2014; Mondal et al., 2017). Cultivated commercial rice on the other hand does not survive beyond 6dS/m (Haque et al., 2022). *Oc* is an allotetraploid with a unique KKLL genome which has evolved through interspecific hybridization and genome doubling (Jacquemin et al., 2013; Liu et al., 2020). However, the majority of diploid progenitors of those genomes are presumed to be extinct (Ammiraju et al., 2010). *Oc*, has distinct physical characteristics in contrast to rice such as upright, closely spaced stems with dense roots and a well-developed underground rhizome system that produces leafy shoots. Rootlets originating from the rhizome help in absorption and anchoring, even under high salinity conditions. The plant’s waxy leaves with narrow, sturdy blades and unique salt glands aid in its growth and survival in constant exposure to salty water and high temperatures (Sengupta and Majumder, 2009; Garg et al., 2014; Mondal et al., 2017). Additionally, *Oc* thrives in submerged conditions, indicating the presence of genes that confer submergence tolerance, similar to those found in cultivated rice varieties. *Oc* reproduces vegetatively since its seeds usually do not germinate. The unbranched panicle of *Oc* produces 10-12 soft grains, which are sometimes used as cattle feed in coastal regions. When compared to rice, *Oc* demonstrated significantly better performance in terms of chlorophyll content, growth, and photosynthetic efficiency under various salt stress conditions (Sengupta and Majumder, 2009).

A novel approach could be investigation of possible synergy between growth of halophytes (salt-loving) in close proximity (paired) to commercial genotypes (salt-sensitive but high-yielding) in the same environment. It was reported that *Oc* depends on Na^+^ ion transfer into *the* shoot for osmotic adjustment (Ishikawa et al., 2022). Therefore, it likely reduces that salinity of the soil or water that it is proximal to and in this process of detoxification, may help a less tolerant plant to grow by its side. The potential danger of gene flow from the wild *Oc* to cultivated *Os* to make the latter weedy (Roma-Burgos et al., 2021), was deemed minimal because forced hybridization between the two did not result in successful outcome (Islam et al., 2017; Maisha et al., 2023).

Any possible mutualism between *Oc* and *Os* may be inclusive of any endophytic microbes growing within the halophyte and helping to defend the commercial but sensitive genotype against the stress. Our earlier work has identified a highly significant plant growth promoting endophytic fungus associated with *Oc* roots and rhizomes (Airin et al., 2023). Apart from that, previous studies showed plants communicate with neighboring plants and modify their physiological and morphological features, including alterations in root system architecture (RSA) (Zhang et al., 2022). Changes in biomass accumulation, horizontal and vertical asymmetrical distribution are the primary behavioral adaptations that occur in response to neighboring plants (Zhang et al., 2022).

Habitat-adapted symbiosis in adverse environments is often achieved by forming associations with bacterial endophytes. Such naturally adaptive bacterial endophytes may be one of the factors in mitigating the effect of such excessive salinity stress. Previous studies showed these beneficial microbes have the ability to positively impact plant growth and metabolism in various stressful environmental conditions through the activation of essential genes, enzymes, metabolites, and hormones (Bloemberg and Lugtenberg, 2001; Yang et al., 2009; Naher et al., 2018). For example, this is achieved by facilitating complex phosphate solubilization, and improving photosynthesis and water use efficiency (Hamdali et al., 2008; Porcel et al., 2012; Yadav, 2020). Apart from this, some may mitigate abiotic stresses by producing antioxidant enzymes like catalase, superoxide dismutase (SOD), and peroxidase and by emitting volatile substances that positively influence plant growth (Dimkpa et al., 2009; Timmusk et al., 2014; Islam et al., 2016).

In this study, we established a co-growth experiment between *Oc* and *Os* plants to observe the effect of the halophyte, if any, to help *Os* plants survive in a saline environment and perform better compared to the non-paired condition. Although the *Os* genotype that we used is moderately tolerant, the objective was to check whether it can tolerate even higher salt in the presence of *Oc.* Additionally, we identified the endophytic microbial community in the roots of *Oc and Os* both in control and saline condition from the pairing experiment. Finally, we performed a comparative RNA-Seq based transcriptome analysis with an emphasis on determining how paired *Os* plants fared in high salt stress compared to the unpaired condition. RNA-Seq is a transcriptome profiling method that employs advanced sequencing technologies for deep sequencing (Wang et al., 2009). Differential gene expression between the paired and unpaired condition under control and stress was analyzed to unravel any key genes and pathways related to synergy between the rice and its salt-loving distant relative. Previous RNA-seq work has identified regulatory genes such as transcription factors and protein kinases involved in interactions between non-legumes and beneficial bacteria which can have long-term implications towards sustainably improving agriculture (Thomas et al., 2019). Likewise, the expression of a few selected differentially expressed genes (DEGs) were evaluated and validated by quantitative RT-PCR (qRT-PCR). We could clearly demonstrate the benefits of the pairing of *Oc* on *Os in* normal and salty conditions. Therefore, such a methodology can be used to improve cultivation practices for saline soils.

## 2 Materials and methods

### 2.1 Establishment of a mutualism experiment

**Figure 1:**
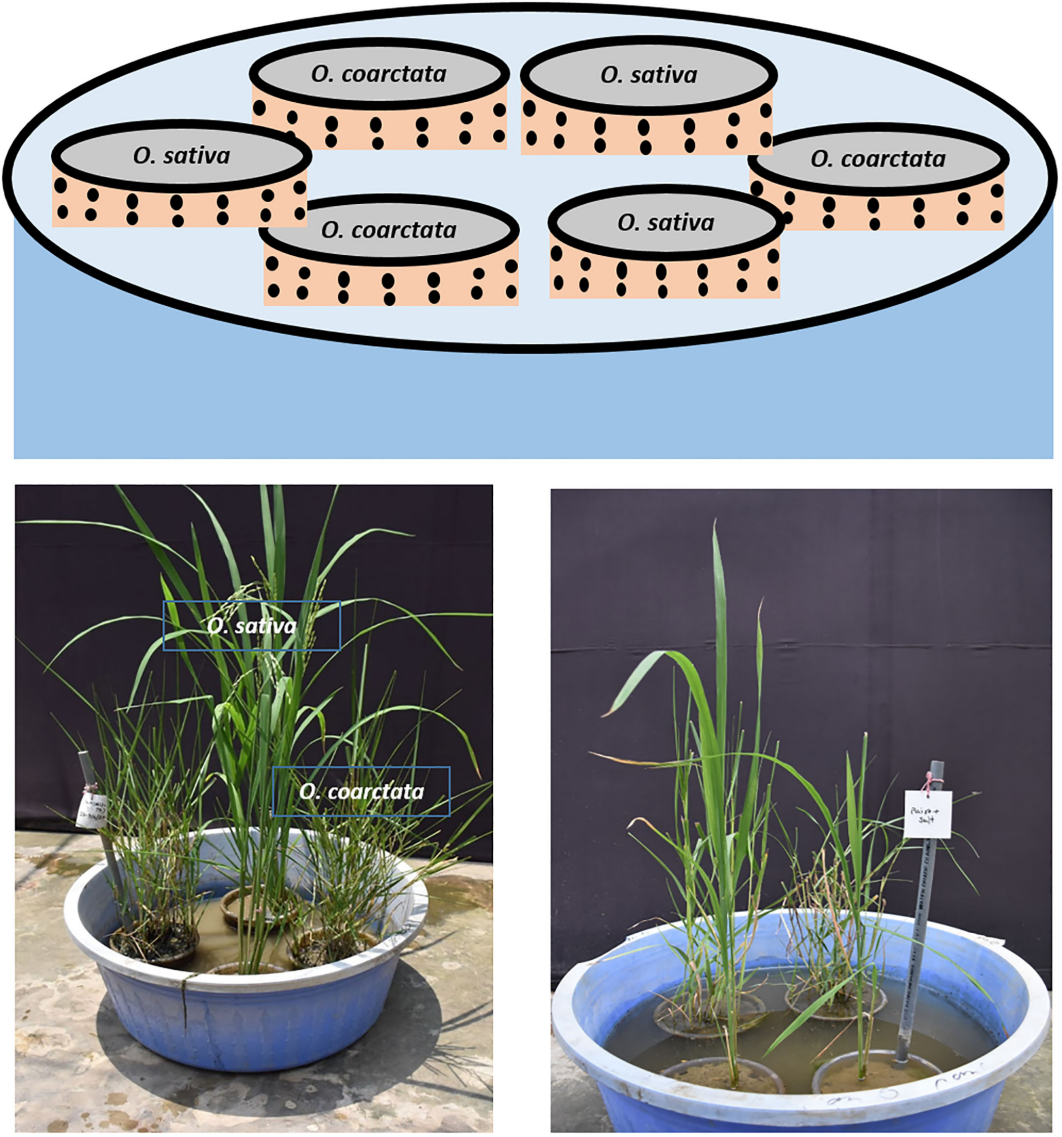
Graphical and experimental representation of pairing experiment which was established in a net house at the University of Dhaka.

We chose the highly salt tolerant wild rice *Oryza coarctata (Oc)*, locally known as Uri dhan and a high yielding commercial rice variety BRRI Dhan67 (BD67), developed by the Bangladesh Rice Research Institute (BRRI) for the testing of a mutualism experiment. The idea was to grow the commercial and halophytic wild rice together (paired) in the same environment under two conditions, one control without salt and in the other in the presence of 100mM salt (NaCl). We planted the commercial rice and wild rice separately in soil in 6-inch plastic pots with 2 mm diameter holes spaced by 1 cm all over, except the bottom. These pots were placed in a large bowl (capacity 55 liters) which could easily fit 6 pots (**Figure 1**). The bowls were filled with tap water up to the top of the soil contained in the pots (40 liters). Prior to planting the rice seedlings, the pots were kept in water for two weeks allowing the soil to settle. One day before planting, the water was drained for 24 hours and refilled with fresh water. At first, 200 BD67 (*Os*) seeds were placed in a 50°C incubator for 48 hours to break dormancy. Then the seeds were incubated at 37°C for germination. After 4 days the seeds were transferred to a floater containing a hydroponic Yoshida solution (Kim et al., 2005). 96 seedlings were placed against a mesh attached to a styrofoam in 8 liters of the solution. 8 day-old BD67 plants were transferred from the solution to soil in the pots with holes. *Oc* plant propagules collected from the Teknaf coastal area on the east tip of Bangladesh (Islam et al., 2017) containing roots and rhizomes were allowed to grow in the hydroponic Yoshida solution for 3-4 weeks before transferring to the holed pots. The pots of *Os and Oc* were placed alternately in the bowl described above (paired) filled with water up to the brim of the pots (3 each in one). Thus, the commercial and wild rice were in the same environment connected by water. For comparison there were similar bowls with only *Os* plants (unpaired in 6 holed pots) and bowls only containing *Oc* (unpaired in 6 holed pots). For phenotypic screening in both seedling and reproductive stages, salt stress was applied to a set of bowls by replacing the water with NaCl solution (measured by the conductivity meter) in different time frames. The soil in the pots gets equilibrated with the salt water in a few days and were tested to ensure the desired conductivity (Gregorio et al., 1997). Water in control and salt stressed bowls were topped up every day to maintain the water to the corresponding brim of the pots inside. Phenotypic changes were observed weekly. The conductivity of the water was measured on alternate days. When the effects of the salt stress were visible, samples were collected for analysis as appropriate for measuring the seedling stage and reproductive stage stress effects as explained below.

### 2.2 Screening of plant growth and oxidative stress parameters

#### 2.2.1 Seedling stage

When the age of the plants were 19 days after emergence and at the four leaves stage, the water in the bowls was adjusted to 40 mM with NaCl and gradually increased by increments of 20mM NaCl equivalents to 100mM at 24 hr intervals (as measured by the conductivity meter, which showed an increase of 2 dS/m after each increment). An equal number of control bowls were used with water only. After 72 hr of 100mM salt stress when the effect of salt stress was visible, root and leaf samples were collected for physiological and morphological parameter measurements like shoot length, root length, shoot weight, root weight, chlorophyll content, sodium and potassium content, hydrogen peroxide content and electrolyte leakage. The chlorophyll content of leaves was determined using the following procedure: a leaf was cut into small pieces measuring approximately 1 cm² and weighed. The leaf pieces were then soaked in 20 mL of 80% acetone in a dark environment. After 48 hours, the absorbance of the solution was measured at wavelengths of 645 nm for chlorophyll a, 663 nm for chlorophyll b, and 652 nm for total chlorophyll (Chutia and Borah, 2012).

To measure the sodium (Na^+^) and potassium (K^+^) concentrations in leaves at the seedling stage, the plants were first washed with flowing tap water. The leaves were then dried in an oven. After drying, the leaves from each replicate were combined, ground, and subjected to analysis using a flame photometer (Sherwood model 410, Sherwood, UK) following a 48-hour extraction with 1 N hydrochloric acid (HCl). The concentrations of Na^+^ and K^+^ were expressed in millimoles per gram of dry weight.

#### 2.2.2 Reproductive stage

30 days after transplanting and at least 10 days prior to panicle initiation the water in the bowls was adjusted to 100mM NaCl. Phenological parameters were measured after the seeds matured, about 70 days after addition of the salt. An equal number of control bowls were used with water only. Spikelet fertility, panicle length, flag leaf length, 1000-grain weight (GW), total GW, grain yield, average panicle length, total tiller number, effective tiller number, and 1st leaf sodium and potassium content were measured at maturity stage.

All statistical analyses were done using R Studio version 4.2.2. To compare the differences between the paired and unpaired *Os* plant both in control (without NaCl) and saline (100mM NaCl) in the case of seedling stage experiment and reproductive stage conditions, the analysis of paired T test was performed using conditions and individual plants as factors. Values of p < 0.05, p < 0.03, p < 0.01, and p < 0.001 were deemed significant, moderately significant, highly significant, and very highly significant respectively. All the graphical representations were prepared using the ggplot2 package.

### 2.3 Endophytic bacteria isolation and molecular identification

#### 2.3.1 Isolation

The 29 days-old mutually grown BD67 (*Os*) roots and *Oc* roots and rhizomes samples were collected for endophyte isolation. For isolating endophytic bacteria, a protocol as described by Anjum and Chandra (2015) was followed with slight modification. The protocol was optimized by adjusting the time of washing the rhizomes/roots. To isolate endophytic bacteria, plants were uprooted carefully without damaging the rhizomes/roots. Intact rhizomes/roots were washed first with tap water for 15 minutes to remove the attached soil. Then the rhizomes/roots were washed with 70% ethanol for 1 minute and 2% sodium hypochlorite for 1 minute and 30 seconds. This was followed by washing 5 times with autoclaved ddH_2_O. Water from the final wash was kept as a negative control. The surface sterilized rhizomes/roots were cut into small pieces and crushed separately for each sample in a phosphate buffer of pH 7.2 with a sterile mortar and pestle. Tissue extracts were then prepared for tenfold dilution in the sterile phosphate buffer. For inoculation, 0.1 ml of the aliquot was used on a Tryptone soy agar (TSA) medium. All the diluted and undiluted samples were also spread on different TSA plates including the negative control. All plates were then incubated at 37°C for 24 hours. After incubation, bacterial colonies were differentiated on the basis of colonial characteristics. For isolation as pure culture, the bacterial isolates from the distinctly differentiated colonies were picked up from the culture plates and sub cultured on fresh TSA plates by streak-plate technique.

#### 2.3.2 Molecular identification

We isolated the total genomic DNA from our chosen halotolerant bacteria by subjecting the bacterial cells to lysis. Subsequently, we quantified the DNA using a Nanodrop spectrophotometer (ND-1000) and confirmed the integrity by gel electrophoresis on 0.8% agarose gel. To study a specific region of the genomic DNA that encodes the 16S rRNA gene, we performed PCR amplification. We used a pair of conserved primers, F1 (GAGAGTTTGATCCTGGCTGGCTCAG) and R1 (AAGGAGGTGATCCAGCCGCA), which approximately amplified 1500 base pairs of the gene. The PCR reactions included 100 ng of genomic DNA, 100 μmol/L of each dNTP, 2.4 ng of each primer, 1 unit of Taq Polymerase, 1× PCR buffer (without MgCl_2_), 1.5 mmol/L MgCl_2_, and 2.4% DMSO from Invitrogen, USA. The amplification process started with an initial denaturation at 95°C for 5 minutes. This was followed by 35 cycles of three steps: denaturation at 95°C for 30 seconds, annealing at 60°C for 40 seconds, and extension at 72°C for 50 seconds. A final extension step at 72°C for 10 minutes was performed using a thermocycler called GeneAtlas of Astec. The amplified PCR products were then separated on a 1.0% agarose gel. We confirmed the presence of the desired DNA bands by comparing them to a 1 Kb^+^ DNA ladder as a size reference (Singh et al., 2016; Vimal et al., 2019). Following gel confirmation, we sent the purified DNA to Apical Scientific in Malaysia for sequencing. The nucleotide sequencing was performed using the Sanger dideoxy method. The resulting nucleotide sequences were manually edited and compared to the GenBank database using the NCBI BLAST algorithm (www.ncbi.nlm.nih.gov/BLAST) for further analysis and identification of the bacteria.

### 2.4 RNA extraction, library construction and Illumina sequencing

At the seedling stage, after 72 hr of 100mM salt stress and when the effect of salt stress was visible, 100-200mg of the leaves of both control and treated samples were directly flash-frozen in liquid nitrogen and stored at −80°C until RNA extraction. Total RNA was extracted using Tri-RNA reagent (Favorgen, Kaohsiung, Taiwan) following the manufacturer’s procedure. To analyze the quantity and purity of total RNA, the Bioanalyzer 2100 and the RNA 6000 Nano Lab Chip Kit from Agilent (CA, USA) were utilized. The RNA samples were assessed, and only those with a RIN (RNA Integrity Number) above 7.0 were selected. For RNA sequencing (RNA-seq) analysis, three replicated samples from each condition were pooled together and the pooled RNA samples were then sent to LC Sciences, 2575 West Bellfort Street Suite 270 Houston, TX 77054 USA (https://lcsciences.com/) for further processing, including library construction, sequencing, and preliminary bioinformatics analysis. Approximately 5 μg of total RNA was used for mRNA purification using the Ribo-Zero Gold rRNA Removal Kit from Illumina (cat.MRZG12324, San Diego, USA). The remaining RNA molecules were fragmented into shorter fragments using the NEBNext Magnesium RNA Fragmentation Module (cat.E6150S, USA), which employs divalent cations under high temperatures. The fragmented RNA was then reverse-transcribed using SuperScript II Reverse Transcriptase from Invitrogen to generate cDNA. This cDNA was used to synthesize second-stranded DNAs labeled with U using E. coli DNA polymerase I (NEB, cat.m0209, USA), RNase H (NEB, cat.m0297, USA), and dUTP Solution (Thermo Fisher, cat.R0133, USA). The resulting double-stranded cDNAs were purified and subjected to end repair, dA tailing, adaptor ligation, and DNA fragment enrichment. The final cDNA library had an average insert size of 300 ± 50 bp. Lastly the cDNA library was subjected to paired-end sequencing (2×150 bp) on an Illumina Novaseq 6000 platform, following the recommended protocol provided by the vendor.

Illumina reads of all samples have been submitted to the at the National Center for Biotechnology Information (http://www.ncbi.nlm.nih.gov/sra) under bioproject accession number PRJNA982590.

### 2.5 RNA-seq data processing and differential gene expression analysis

Initially, Cutadapt (Martin, 2011) and custom Perl scripts were employed to remove reads containing adaptor contamination, low-quality bases, and undetermined bases. The quality of the resulting clean data was assessed using FastQC, which included analysis of Q20, Q30, and GC-content (Anders and Huber, 2010). For read mapping to the reference genome, HISAT2 (https://daehwankimlab.github.io/hisat2/, version: hisat2-2.0.4) was utilized. The reference genome was obtained from the NCBI database (https://ftp.ncbi.nlm.nih.gov/genomes/all/GCF/001/433/935/GCF_001433935.1_IRGSP-1.0). HISAT2 allows multiple alignments per read (up to 20 by default) and permits a maximum of two mismatches during mapping. It builds a database of potential splice junctions and validates them by comparing unmapped reads against the database (Pertea et al., 2016).

Following read mapping, StringTie (Pertea et al., 2015) was employed to assemble the mapped reads for each sample. Perl scripts and gffcompare (http://ccb.jhu.edu/software/stringtie/gffcompare.shtml, version: gffcompare-0.9.8) were used to merge all transcriptomes from the experimental samples and reconstruct a comprehensive transcriptome. Once the final transcriptome was generated, expression levels of all transcripts were estimated using StringTie (Pertea et al., 2015) and Ballgown (Pertea et al., 2016) packages. The expression levels of mRNAs were calculated using FPKM (Fragments Per Kilobase of transcript per Million mapped reads).

The comparison between two different groups was conducted using DESeq2 software, allowing for the identification of differentially expressed genes. Genes with an FDR below 0.05 and an absolute fold change of ≥2 were considered significant.

To identify significantly enriched gene ontology (GO) terms associated with differentially expressed genes, functional enrichment analysis, including GO analysis, was performed using the Gene Ontology database (http://www.geneontology.org). GO terms with an FDR ≤ 0.05 were considered significantly enriched by the differentially expressed genes. Additionally, Kyoto Encyclopedia of Genes and Genomes (KEGG) pathway analysis was conducted to identify enriched pathways, brite, and modules (Kanehisa et al., 2021).

### 2.6 Quantitative real-time PCR assay

To validate the RNA-Seq data, the expression pattern analysis of specific differentially expressed genes was conducted by quantitative reverse transcription–polymerase chain reaction (qRT-PCR) as we had only pooled samples from replicates selected for total RNA sequencing. As explained in sections 2.1 and 2.2, 19 days old rice seedlings were treated with 40mM salt stress that was gradually increased to 100mM in 24 hr intervals. An equal number of control bowls were used with water only. After 72 hr of 100mM salt stress, root samples of *Os* plants were collected for RNA extraction using the RNeasy Plant Mini kit, QIAGEN. Four genes were retrieved from the Rice Genome Annotation Project (RGAP) with diverse expression profiles, including up-regulated or down-regulated at under different conditions. The primers of the selected genes were designed using primer 3 plus and oligo analyzer software, and the primers used in the qRT-PCR are provided in **Table 1**. A housekeeping gene named small-nucleolar RNA (SNO) was used as an internal control in qRT-PCR. Quantitative Real-time PCR (qPCR) was performed in a 10 μl reaction using Luna^®^ Universal One-Step RT-qPCR Kit (New England Biolabs) in a CFX Opus Real‐Time PCR detection system (Bio‐Rad, USA). PCR efficiency (90-95%) was confirmed and amplification specificity was validated by melt curve analysis of each PCR cycle. The relative expression level of the targeted DEGs were calculated with the 2^-ΔΔCT^ method (Livak and Schmittgen, 2001). The reaction was carried out using three biological replicates along with three technical replicates.

**Table 1:** Primers used for the RT-PCR analysis.

primer name	Sequence	Tm(°C)	GC content (%)	Product length
Auxin_F	CCC TGG GTT TTC CTA CCA TT	62	50	199 bp
Auxin_R	ATC ACA TCC TTG TGG TGC AA	62	45	
ABCG43_F	AAGAGGCTGACAATCGCAGT	60	50	155 bp
ABCG43_R	TGGATTGTGCAAACCACAGT	60	43	
glcABA_F	AAC CAA AAC CGG AGC CCA AA	62	50	187bp
glcABA_R	TTT GGT TCT GGC TTT GGC TCA	62	47.5	
JuB_F	GCAATGGAACAGTAGCAGCA	62	50	171bp
JuB_R	GAGCTCCATCACCATCCTGT	62	45	
Sno RNA_F	ACAGATAAGATTAGCATGGCCCC	60	47.8	160
Sno RNA_R	GGACCATTTCTCGATTTGTACGTG	60	45.8	

## 3 Results

### 3.1 Improved phenotypic response to salt stress

#### 3.1.1 Seedling stages

**Figure 2:**
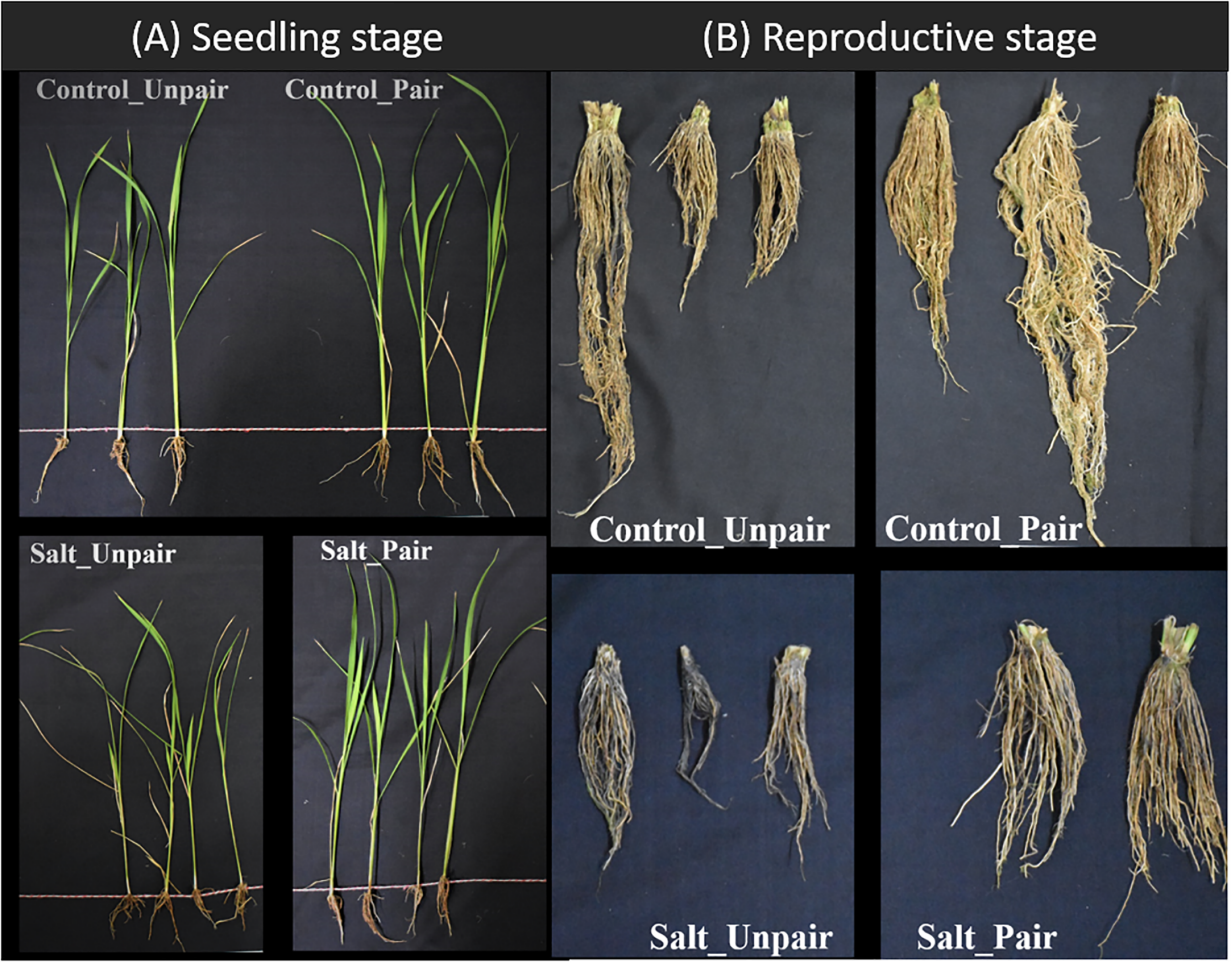
Phenotypic appearance in both seedling **(A)** and reproductive stage **(B)** between paired and unpaired *O. sativa* (BD67) plants under salinity stress.

**Figure 3:**
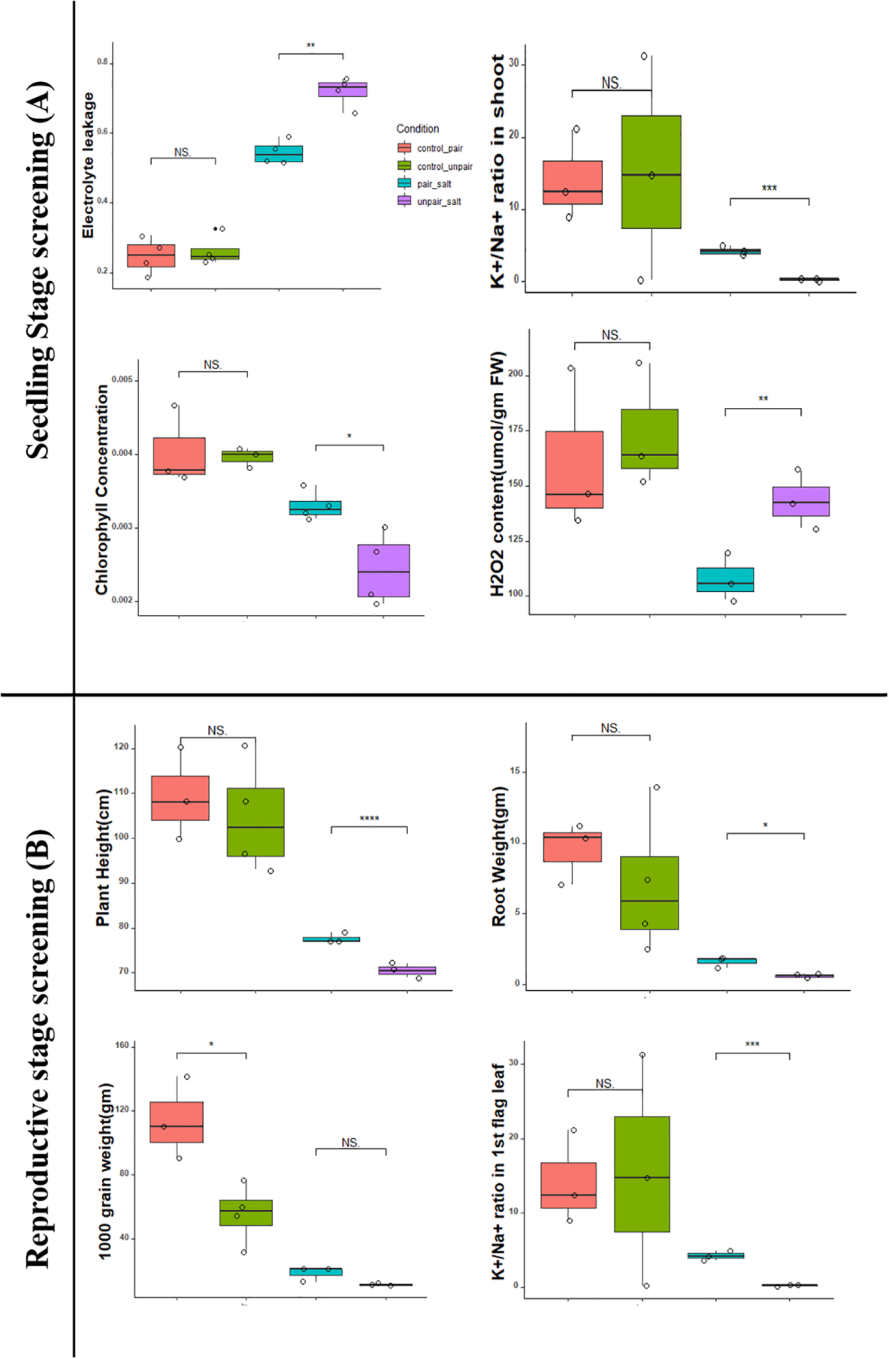
Comparison of pairing effect on both seedling and reproductive stage in both control and treatment of *O. sativa* plants (BD67). Here, **** indicates very highly significant (p< 0.001), *** highly significant (p< 0.01), ** moderately significant (p< 0.03) and * significant (p< 0.05).

The effect of mutualism on commercial rice (BD67, *Os*) under both normal and 100mM salt stress was examined and compared with non-paired (without adjacently grown) wild rice (**Figure 2A, 2B**). Agronomic traits like shoot length, root length, shoot weight, root weight, electrolyte leakage, chlorophyll content, H_2_O_2_ content, Na^+^/K^+^ and K^+^/Na^+^ ratios in both root and shoots were assessed both in control (0mM NaCl) and 100mM (10 dS/m NaCl) saline conditions. Among the 11 morphological and physiological characteristics analyzed, 4 significantly varied between sativa salt paired (Sativa_SP) and unpaired *Os* (Sativa_SUp) plants under salinity stress. It was observed that the shoots of paired *Os* plants in stressed condition showed significantly less (p<0.03) electrolytic damage and a significantly increased (p<0.03) chlorophyll content compared to the non-paired condition. At 100mM salt stress, paired *Os* shoots also showed significantly less (p <0.03) H_2_O_2_ content compared to the control (unpaired *Os*) but a highly significant increase in the (p<0.01) K^+^/Na^+^ ratio. Considering all parameters tested, it was clear that paired *Os* (Sativa_CP) seedlings showed significantly better performance compared to non-paired (Sativa_CUp) under both control (0mM NaCl) conditions as well as in 100mM salinity stress (**Figure 3A**).

#### 3.1.2 Reproductive stage

The yield parameters including other agronomic traits like plant height, root length, root weight, total tiller, effective tiller, total grain weight, yield gm per plant, 1000 grain weight, panicle length, Na^+^/K^+ of^ roots, and Na^+^/K^+^ of first leaf were assessed in 140 days old plant both in control (0mM NaCl) and saline 100mM (10 dS/m NaCl) conditions. Among 13 traits, 3 agronomic traits (plant height, average panicle length, K^+^/Na^+^ ratio in 1st flag leaf) showed significantly less reduction in stress-challenged *Os* plants between paired and unpaired conditions. Additionally, 3 traits (total grain weight, yield gm per plant, 1000 grain weight) were significantly increased without salt in the plants under comparison (**Figure 3B**) (Phenotypic data in **Supplementary File 4**).

### 3.2 Endophytic bacterial diversity explored in salt stress mutualism of two rice species

The habitat-adapted endophytic bacteria associated with rhizomes and root of the *Oc* and *Os* plants were considered to be more informative compared to other tissues (stem and leaves), since these are in direct contact with the stress in the soil and will likely be affected first and adjust to the environmental perturbation accordingly. A total of 39 different types of bacteria belonging to 9 different genera were isolated from the pairing experiment (**Table 2**). Among them, 21 bacteria were isolated from *Oc* root and rhizome and the rest from *Os* roots in both control and paired conditions under 0mM salt stress and 100mM salt stress (**Table 2**).

**Table 2:** List of Isolated Endophytic bacteria from mutualism experiment.

Plant_Variety	Condition	Bacterial_species	Bacterial_Genus
BRRIDhan67	Sativa_Control Unpair	*Bacillus australimaris*	*Bacillus*
BRRIDhan67	Sativa_Control Unpair	*Bacillus proteolyticus*	*Bacillus*
BRRIDhan67	Sativa_Control Unpair	*Bacillus megaterium*	*Bacillus*
BRRIDhan67	Sativa_Control Unpair	*Microbacterium paraoxydans*	*Microbacterium*
BRRIDhan67	Sativa_Control pair	*Achromobacter insuavis*	*Achromobacter*
BRRIDhan67	Sativa_Control pair	*Paraburkholderia EVS-6*	*Paraburkholderia*
BRRIDhan67	Sativa_Control pair	*Bacillus marisflavi*	*Bacillus*
BRRIDhan67	Sativa_Salt unpair	*Achromobacter insuavis*	*Achromobacter*
BRRIDhan67	Sativa_Salt unpair	*Paraburkholderia phytofirmans*	*Paraburkholderia*
BRRIDhan67	Sativa_Salt unpair	*Paraburkholderia EVS-11*	*Paraburkholderia*
BRRIDhan67	Sativa_Salt unpair	*Bacillus licheniformis*	*Bacillus*
BRRIDhan67	Sativa_Salt unpair	*Bacillus aryabhattai*	*Bacillus*
BRRIDhan67	Sativa_Salt unpair	*Bacillus tropicus*	*Bacillus*
BRRIDhan67	Sativa_Salt pair	*Aeromonas caviae*	*Aeromonas*
BRRIDhan67	Sativa_Salt pair	*Pseudomonas otitidis*	*Pseudomonas*
BRRIDhan67	Sativa_Salt pair	*Paraburkholderia fungorum*	*Paraburkholderia*
BRRIDhan67	Sativa_Salt pair	*Bacillus licheniformis*	*Bacillus*
BRRIDhan67	Sativa_Salt pair	*Bacillus aryabhattai*	*Bacillus*
*Oryza coarctata*	Control_Unpair	*Cellulomonas hominis*	*Cellulomonas*
*Oryza coarctata*	Control_Unpair	*Achromobacter xylosoxidans*	*Achromobacter*
*Oryza coarctata*	Control_Unpair	*Achromobacter insuavis*	*Achromobacter*
*Oryza coarctata*	Control_Unpair	*Bacillus marisflavi*	*Bacillus*
*Oryza coarctata*	Control_Unpair	*Bacillus aryabhattai*	*Bacillus*
*Oryza coarctata*	Salt_unpair	*Aeromonas dhakensis*	*Aeromonas*
*Oryza coarctata*	Salt_unpair	*Aeromonas veronii*	*Aeromonas*
*Oryza coarctata*	Salt_unpair	*Achromobacter insuavis*	*Achromobacter*
*Oryza coarctata*	Salt_unpair	*Bacillus altitudinis*	*Bacillus*
*Oryza coarctata*	Salt_unpair	*Bacillus aryabhattai*	*Bacillus*
*Oryza coarctata*	Salt_unpair	*Microbacterium paraoxydans*	*Microbacterium*
*Oryza coarctata*	Control_Pair	*Achromobacter insuavis*	*Achromobacter*
*Oryza coarctata*	Control_Pair	*Bacillus tropicus*	*Bacillus*
*Oryza coarctata*	Control_Pair	*Bacillus marisflavi*	*Bacillus*
*Oryza coarctata*	Control_Pair	*Bacillus stratosphericus*	*Bacillus*
*Oryza coarctata*	Control_Pair	*Staphylococcus warneri*	*Staphylococcus*
*Oryza coarctata*	salt_pair	*Aeromonas media*	*Aeromonas*
*Oryza coarctata*	salt_pair	*Achromobacter insuavis*	*Achromobacter*
*Oryza coarctata*	salt_pair	*Oerskovia paurometabola*	*Oerskovia*
*Oryza coarctata*	salt_pair	*Bacillus xiamenensis*	*Bacillus*
*Oryza coarctata*	salt_pair	*Bacillus tropicus*	*Bacillus*

**Figure 4:**
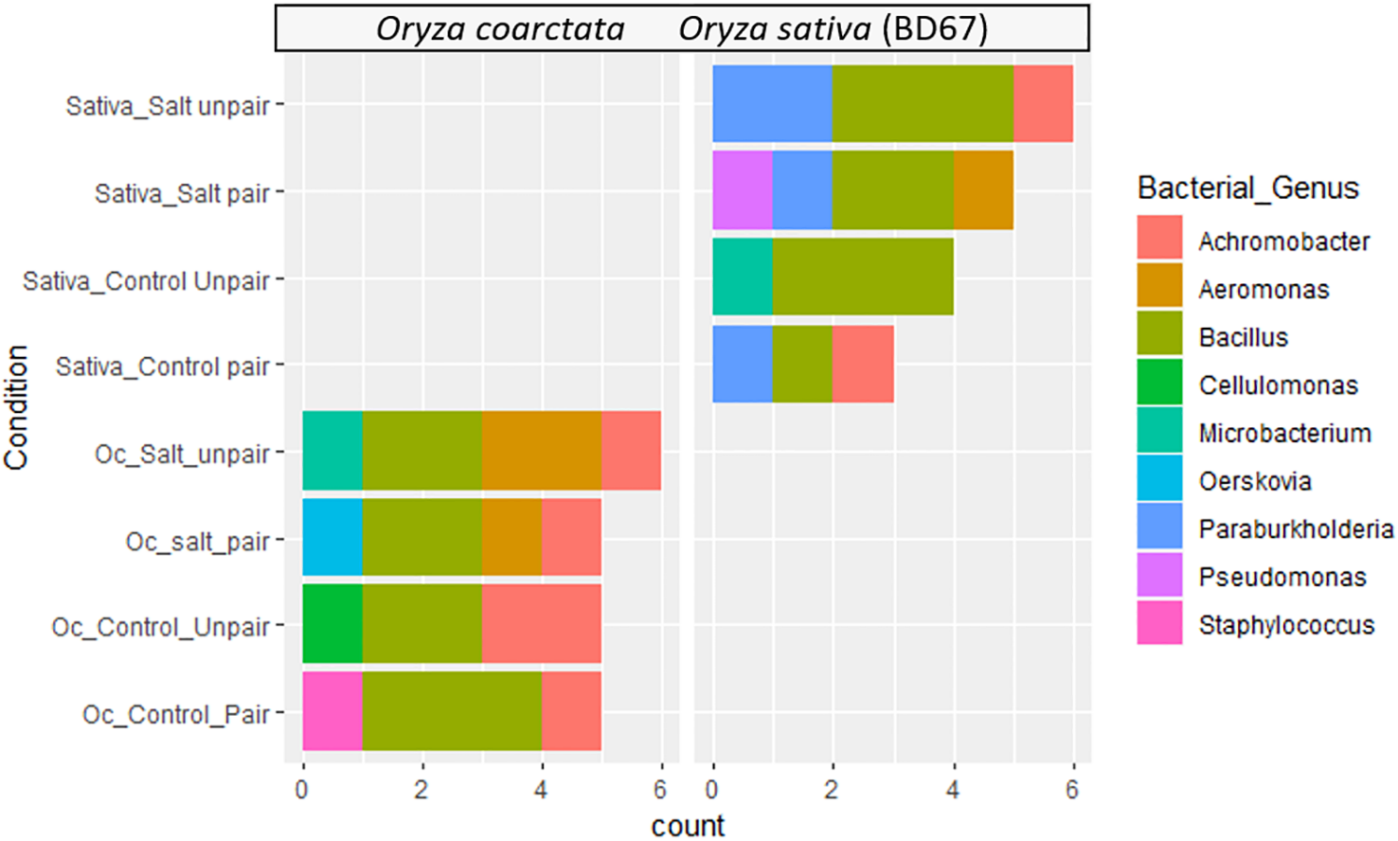
Distribution of endophytic bacterial communities in different experimental groups and conditions based on 16s rRNA sequences.

Diverse bacterial endophytes were found both in wild and commercial rice collected from the pairing experiments. The relative abundances of the genus of bacterial endophytes of the plants showed considerable variation at the genus level (**Figure 4**). Bacillus spp. was found to be the most abundant bacterial endophyte of both plant species. However, Paraburkholderia spp. was only found in *Os* as the second abundant genus whereas Oerskovia was found uniquely in *Oc*. Additionally, Pseudomonas spp. only appeared in paired *Os* under salt stress. Aeromonas and Achromobacter were not present in unpaired *Os* but were found in *Oc*. Interestingly, Aeromonas was present in *Oc* only under salt. Both these genera were transferred to *Os* from *Oc* in paired conditions, Achromobacter without stress and Aeromonas under stress. Therefore, bacteria do seem to affect the commercial genotype in mutualism experiments.

We conducted a study to analyze several specific endophytes found in *Oc* that exhibited growth-promoting properties when subjected to high salinity stress. These endophytes were observed to possess characteristics such as zinc solubilization, phosphate solubilization, and nitrogen fixation (**Supplementary Table 1**). The methodology for this characterization was not provided; however, their growth properties have been detailed in **Supplementary Table 1**.

### 3.3 Mutualistic growth with Oryza coarctata results in a distinctive pattern of differential gene expression in Oryza sativa

In the pairing experiment, shoot samples from both wild and commercial rice were selected for RNA-Seq analysis. The total RNA extracted from these shoot samples was used to create cDNA libraries. The quality of the raw reads was evaluated using FastQC, followed by the removal of adapter sequences and low-quality reads. The clean reads obtained ranged from 73,747,382 to 80,548,614 (**Supplementary File 1**); and the available clean reads were mapped to the reference Japonica genome using HISAT. The mapping data revealed that 81.84 to 91.80% of the reads were successfully mapped to the Japonica reference genome in case of *Oryza sativa* shoots whereas only 58.17 to 66% were mapped to the Japonica reference genome in the case of *Oryza coarctata*. To investigate the significance of gene expression differences between experimental factors, the transcript abundance of each gene was normalized to the FPKM value.

**Figure 5:**
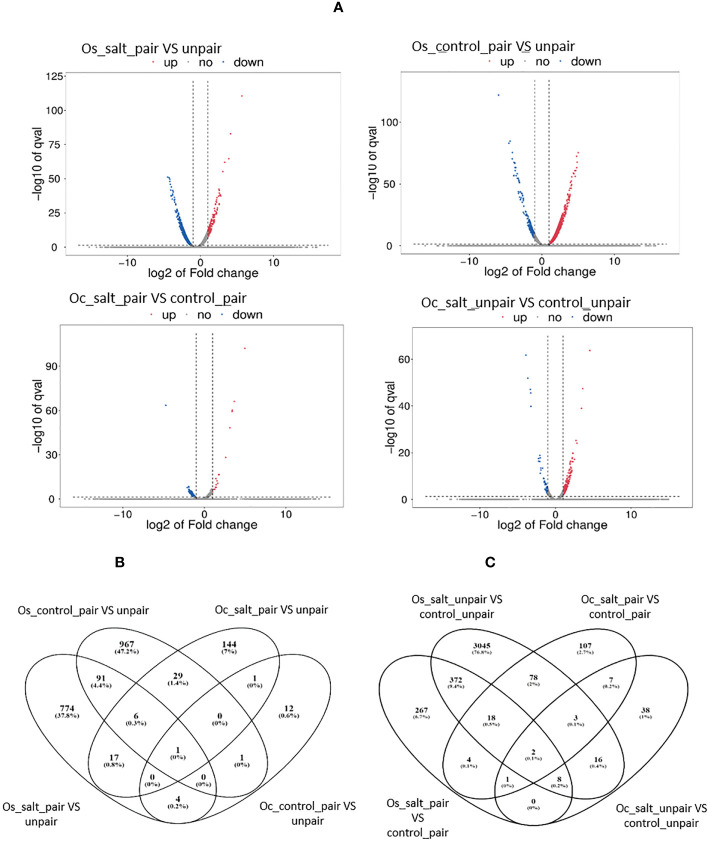
Volcano plot and Venn Diagram of Differentially expressed genes from both *O. sativa* and *O. coarctata* during mutualism under salinity stress. **(A)** The x-axis represents fold change (Fc) and the y-axis represents negative log10 of the q-value of each gene. In general, *Oryza sativa* showed stronger signals in leaves tissue compared to *Oryza coarctata*. **(B)** the Number of unique and overlapping genes between salt and control conditions in both *O*. *sativa* and *O*. *coarctata*. In general, *Oryza sativa* and *Oryza coarctata showed* lower numbers of unique genes. **(B)** the Number of unique and overlapping genes between paired and unpaired condition in both *O. sativa* and *O. coarctata*.

Differentially expressed genes (DEGs) were identified considering the factors of the experimental design and comparison groups based on their pairwise interactions. Significant DEGs were observed for all experimental factors (**Table 3**, **Figure 5**) and in both plant species, with strong DEG signals in *Oryza sativa*. The mutualism effects were prominent for *Oryza sativa* without salt as 994 genes were upregulated and 101 genes were downregulated. Under salinity treatment, 130 genes were upregulated and 763 genes were downregulated for the paired condition. When salinity treatment was considered as the factor for differential expression, 461 genes were upregulated and 261 downregulated for paired plants (Os) compared to paired control whereas in case of unpaired sativa plants, higher number of genes (3453) were upregulated and only 91 genes downregulated.

**Table 3:** Number of significantly differentially expressed genes based on FDR cutoff >=0.05 for Mutualism and Treatment factors.

Rice Variety	Factors	Comparison	Number of DEGs in leaf samples (down and up regulated)
*Oryza sativa*	Mutualism	Control_paired vs Control_unpair	101↓ 994↑
		Salt_pair vs Salt_unpair	763↓ 130↑
	Treatment	Pair_salt vs Pair_Control	261↓ 461↑
		Unpair_salt vs Unpair_Control	91↓ 3453↑
*Oryza coarctata*	Mutualism	Control_paired vs Control_unpair	16↓ 19↑
		Salt_pair vs Salt_unpair	182↓ 16↑
	Treatment	Pair_salt vs Pair_Control	210↓ 10↑
		Unpair_salt vs Unpair_Control	61↓ 226↑

Here ‘Mutualism’ indicates paired and unpaired conditions between two rice species and ‘Treatment’ indicates 0mM and 100mM salinity stress conditions.

Fewer genes were shown to be differentially expressed in *Oryza coarctata* plants as a lower number of genes mapped to the reference genome. In control-paired condition only 19 genes were upregulated and 16 genes were downregulated compared to unpaired-control condition whereas 16 genes were upregulated and 182 genes downregulated in paired salt stress compared to unpaired salt stressed plants. Since our objective was to find how the wild halophyte affected commercial rice under salt stress, mostly the RNAseq results of paired *Os* versus (vs) unpaired was analyzed and discussed further, below.

We found that 98 genes were commonly expressed in both control and salt paired vs unpaired in *O. sativa*. On the other hand, in *O. coarctata*, only 2 genes were common. Interestingly, in control and salt paired conditions for both *O. sativa* and *O. coarctata*, 4 common genes were found to be differentially expressed which were involved in photosynthesis and transportation i.e., photosystem II polypeptide (LOC_Os07g05360), cytochrome P450 (LOC_Os08g43390.1), ABC transporter G family member 43 isoform (LOC_Os07g33780) and transcription factor bHLH63 (LOC_Os03g07510). The latter acts as a positive regulator of grain size.

72 differentially expressed genes in the *O. sativa* control paired vs unpaired were significantly upregulated whereas some among these same genes were downregulated in salt paired vs unpaired plants (**Supplementary Figure 1**). Among these were: similar to phenylalanine ammonia-lyase (OS02G0626100), proline-rich glycoprotein or extensin (OS05G0227600), delta-tonoplast intrinsic protein (OS06G0336200), similar to beta-galactosidase precursor (EC 3.2.1.23) (Lactase) (OS06G0573600) genes, etc. These genes are mostly related to enriched protein storage vacuole, water transport, fluid transport, etc.

Interestingly, we also found 9 differentially expressed genes in paired *O. sativa* which were downregulated without salt but under salt stress, these were upregulated. These 9 genes are mostly hypothetical proteins i.e., hypothetical protein SETIT, hypothetical protein BAE44, hypothetical protein GQ55, peptidyl-prolyl cis-trans isomerase, proline-glutamic acid- and leucine-rich protein 1-like, protein pectic arabinogalactan synthesis-related, etc. These results indicate that in pairing control conditions, these 9 genes were likely involved in housekeeping functionality. However, after salt exposure, these 9 genes were recruited and upregulated for defense under stress in *O. sativa*.

### 3.4 Functional gene enrichment analysis in paired *O. sativa* plants

**Figure 6:**
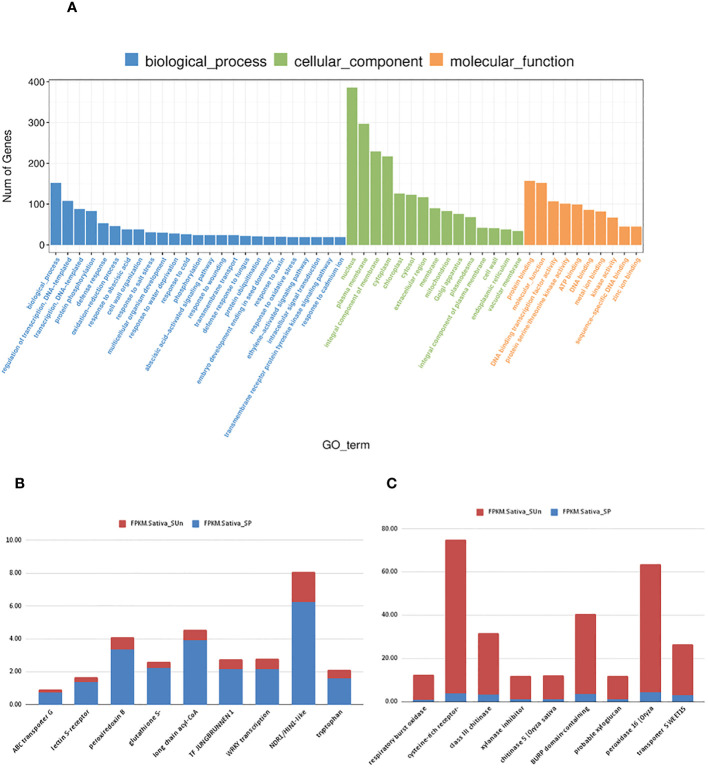
**(A)** Gene ontology analysis in Sativa salt paired vs Sativa salt unpaired plants. **(B)** the Top 10 significantly upregulated genes in Sativa salt paired vs Sativa salt unpaired condition based on FPKM value. **(C)** the Top 10 significantly downregulated genes in Sativa salt paired vs Sativa salt unpaired conditions based on FPKM value. X axis shows the name of genes and Y -axis shows the FPKM values where red indicates the FPKM value of Sativa salt unpaired and blue indicates the FPKM value of Sativa salt paired.

To identify possible biological processes that were altered in paired and unpaired plants under salt-stress, Gene Ontology (GO) and KEGG pathway were performed using an FDR adjusted to p< 0.05 as cutoff (**Figure 6A**). In our study a total 230 GO terms were identified under salt treatment where log2Fc greater than or equal to 1.5 was taken into consideration (**Figure 5A**, **Supplementary File 2**). Our analysis revealed that these DEGs were enriched in response to stress (GO: 0006950), oxidative stress (GO: 0006979), transmembrane transport (GO: 0055085), vacuolar membrane (GO: 0005774), chitinase activity (GO: 0004568), phenylalanine catabolic process (GO: 0006559), protein phosphorylation (GO: 0006468) and ABA signaling pathway (GO: 0009730) (**Figures 5B, C**). In our study, we focused on the significantly enriched top 9 upregulated and downregulated DEGs (**Figures 6B, C**). ABC transporter G family, peroxiredoxin B, glutathione S transferase, TF JUNGBRUNNEN1, WRKY, NDR1/H1N1-like were upregulated while respiratory burst oxidase homolog protein B (LOC_Os12g35610), cysteine-rich receptor-like protein kinase 6 (LOC_Os04g56430), peroxidase 16(LOC_Os06g48030, bidirectional sugar transporter SWEET15 (LOC_Os02g30910) and xylanase inhibitor protein 1-like (LOC_Os11g47600 were downregulated under salt treatment in the *O. sativa* paired group.

**Figure 7:**
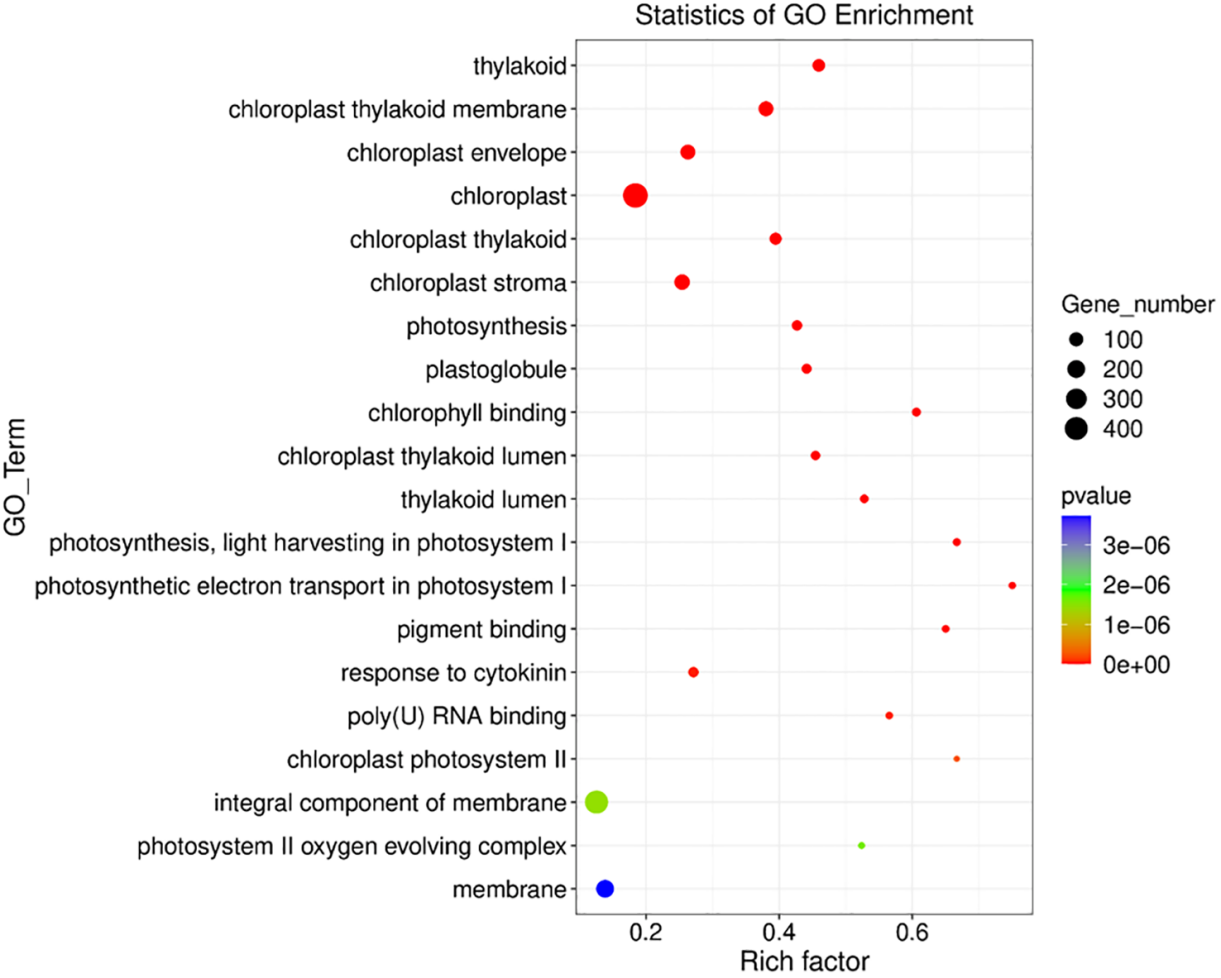
Gene ontology analysis of Sativa control paired vs Sativa control unpaired plants. X axis shows the rich factor of gene ontology and the Y axis shows the overall GO_term.

In control paired vs unpaired, we found 350 GO terms enriched in *Os* plants including biological process (GO terms=185), cellular component (GO terms=52), Molecular function (GO terms=113) (**Figure 7**) (**Supplementary File 3**). Among the 350 GO terms, our analysis revealed that in control paired conditions the significantly expressed DEGs were highly enriched in response to reactive oxygen species (GO: 0000302, 8 genes), photosynthesis (GO: 0015979, 34 genes), response to cytokinin (GO: 0009735, 37 genes), response to phenylalanine (GO: 0080053, 3 genes), ion transmembrane transport (GO: 0034220, 12 genes), nitrate assimilation (GO: 0042128, 11 genes) and regulation of meristem growth GO: 0010075, 8 genes), etc. Interestingly, 56 genes were responsible for the enrichment of response to salt stress (GO: 0009651) under no salt stress (**Supplementary File 2**).

### 3.5 Functional pathways induced by mutualism in paired *O. sativa* plants

**Figure 8:**
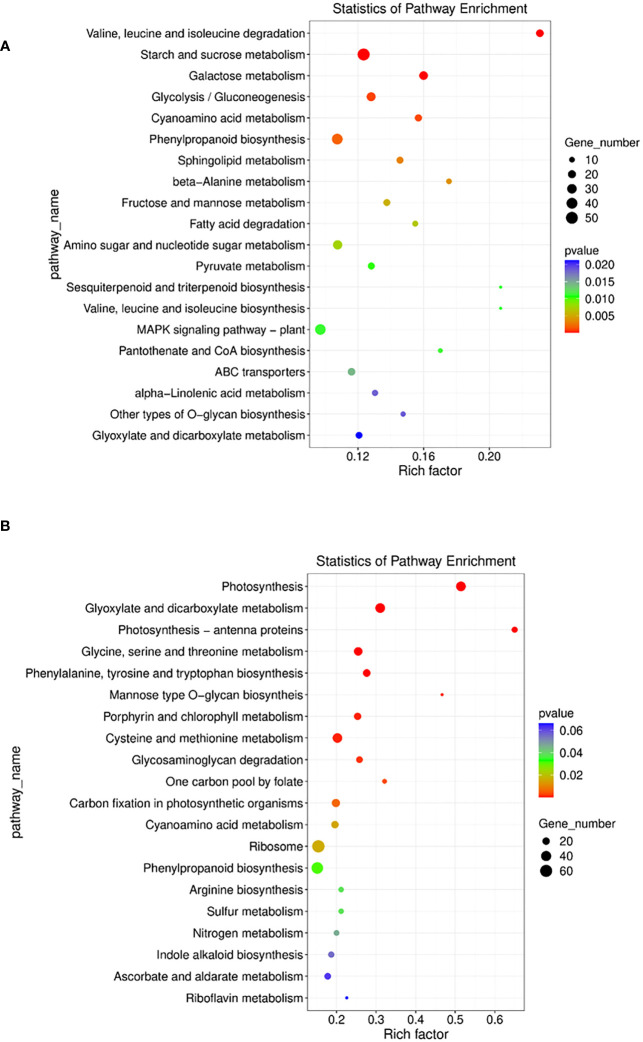
**(A)** E the nriched pathway with statistically significant results from Kegg pathway analysis in Sativa salt paired vs Sativa salt unpaired condition. **(B)** Enriched pathways with statistically significant results from Kegg pathway analysis in Sativa control paired vs Sativa control unpaired condition.

To identify the biological pathways which were enriched by mutualism under salinity stress, the annotated DEGs were mapped to the reference pathways in the KEGG database (Kanehisa et al., 2021). It was found that in the paired condition 73 KEGG pathways, 29 brite and 41 modules were significantly enriched. The most enriched pathways included biosynthesis of cofactors (osa01240), amino sugar and nucleotide sugar metabolism (osa00520), plant hormone signal transduction (osa04075), phenylpropanoid biosynthesis (osa00940), etc. (**Figure 8A**). Apart from that, the MAPK signaling pathway (osa04016) was induced by 3 DEGs and Tryptophan metabolism (osa00380) was also induced by 3 genes (**Supplementary Figure 6**). The differentially expressed auxin-responsive protein IAA18 (osa4339365) gene triggers the plant hormone signal transduction pathway and is responsible for cell enlargement and plant growth (**Supplementary Figure 7**).

Additionally, 3 differentially expressed genes in paired salt stress triggered MAPK signaling pathway whereas WRKY transcription factor 22 (osa9267508) involved in respiratory cell death was downregulated in comparison with unpaired salt stress plants.

Without salt stress, mutualism in the *Os* plant enriched 60 biological pathways, 27 brite and 29 modules as shown by KEGG pathway analysis. The most enriched pathways included photosynthesis, endocytosis (osa04144), mRNA surveillance pathway (osa03015), plant hormone signal transduction (osa04075), phenylpropanoid biosynthesis (osa00940), etc. (**Figure 8B**) (**Supplementary File 3**).

### 3.6 Distinctive carbohydrate metabolism in *O. sativa* shoots in response to mutualism

The strikingly interesting features of the GO terms specifically enriched in the salt paired vs unpaired in comparison to the control paired vs unpaired was the relatively high number of GO terms involved in carbohydrate metabolism. These GO terms involved “glycolysis” (GO: 0006096), “polysaccharide binding process” (GO: 0030247), “carbohydrate biosynthetic process (GO: 0016051” and “lignin catabolic process” (GO: 0046274).

To inclusively assess the biological functions of the DEGs, these were mapped to KEGG database terms with the goal of identifying significantly enriched metabolic or signal transduction pathways. Consistent with the GO enrichment analysis, pathways involved in carbohydrate metabolism, such as “starch and sucrose metabolism”, “fructose and mannose metabolism”, “glycolysis/gluconeogenesis”, and “lignin biosynthesis” were identified and found to be downregulated in the salt paired vs unpaired, while no obviously enriched metabolic pathways were detected in the control paired vs unpaired comparison, with the exception of “lignin biosynthesis (GO: 0009809)”. Similar comprehensive results were found for MAPK signaling pathway, ABC transporters and alpha-linolenic acid metabolism, etc. (**Figures 8A, B**) (**Supplementary File 3**).

### 3.7 Validation of RNA-seq data by quantitative real-time PCR

**Figure 9:**
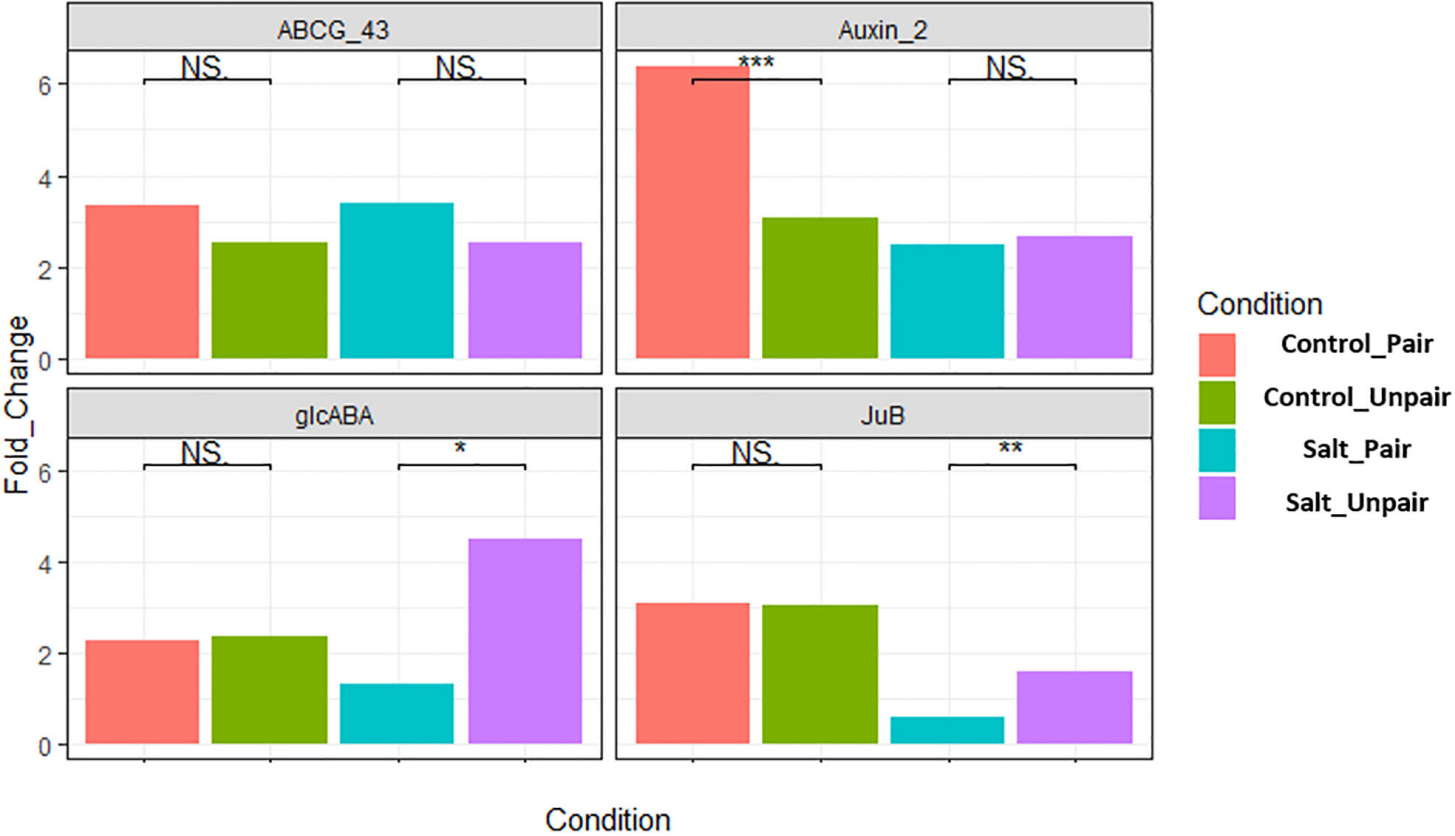
Validation of four differentially expressed genes in *O. sativa* by qRT-PCR. Here, *** indicates highly significant (p< 0.01), ** moderately significant (p< 0.03) and * significant (p< 0.05).

The transcriptome data was verified by conducting RT-qPCR expression analysis on four randomly chosen differentially expressed genes (DEGs) in the leaves of *O. sativa* and were normalized with expression of the respective housekeeping (SnoRNA) gene, LOC112938467 and fixed at a scale of 1.0. These were ABCG43 (LOC4343412), auxin-responsive factor (LOC4325895), glcABA (LOC4338151), JuB (LOC107277389). Among them ABCG43 and JuB were differentially expressed (upregulated) in paired vs unpaired sativa plants under salt stress. The auxin responsive factor 2 gene was upregulated in control paired vs unpaired. glcABA was found to be upregulated in control paired but downregulated in salt paired vs unpaired sativa plants. The findings of RT-qPCR revealed a consistent expression pattern of these genes aligning with the results obtained from the transcriptome analysis (**Figure 9**). The gene JuB was an exception as it is a transcription factor and may change its expression level depending on the time of stress, considering that our RNA sample collection involved separate experiments for qRT-PCR and RNA-seq Therefore, the qRT-PCR analysis confirmed the reliability and accuracy of the transcriptome data and is represented in **Supplementary Figure 8**.

## Discussion

The objective of this study was to investigate if the co-habitation in the same environment (referred to as pairing) of salt sensitive commercial rice with the halophytic wild rice provided any advantages to the former in a preliminary lab-based experiment in terms of growth vigor and yield. The detailed experimental set-up (**Figure 1**) shows that although the commercial rice and wild rice are grown in separate pots these were connected through the shared water, either under control without salt or in 100mM salt. At the seedling stage after approximately 10 days of stress, the *O. sativa* BD67 rice clearly showed better growth under both control and stress conditions when paired with *O. coarctata*. This was in comparison with the BD67 plants without adjacently growing *O. coarctata*. The paired BD67 seedlings performed significantly better under salt stress in terms of having lower electrolyte leakage (p<0.03). Electrolyte leakage can be caused by membrane damage during salt stress which results in reduced plant growth and yield and subsequent death of salt sensitive plants (Demidchik et al., 2014). Reduced electrolyte leakage of paired *Os* plants was indicative of maintenance of high K^+^ in salinity challenged cells and the higher chlorophyll content (p<0.05) showed that photosynthesis was maintained in the plants even at 100 mM salt stress. The membrane damage adversely affects chloroplasts with a concomitant reduction in chlorophyll and photosynthesis (Ali et al., 2004). High chlorophyll, maintenance of the K^+^/Na^+^ ratio (p<0.01) and lower H_2_O_2_ (p<0.03) as observed in our pairing experiments, have consistently been associated with better defense against salt stress (Mishra et al., 2021; Haque et al., 2023). At the reproductive stage, there was lower reduction in height (p<0.001), higher root weight (p<0.05) as well as higher flag leaf K^+^/Na^+^ (p<0.01) in the paired plants under salt stress whereas the differences between salt and no salt was not significant in the unpaired plants. Interestingly, the difference in 1000-grain weight between paired and unpaired was significant without salt, which indicates the positive impact of pairing but not enough to show differences in the yield under salt stress. The positive impact of the pairing with *Oc* may be due to the plant growth promoting bacteria (Sarker et al., 2023) and endophytic fungus associated with *Oc* (Airin et al., 2023).

A question may arise on how to make use of positive impact of growing *Oc* next to cultivated rice in the field. *Oc* is basically a weedy plant and propagates via rhizomes. According to Roma-Burgos et al. (2021), there is a danger of de-domestication of cultivated rice due to the gene flow from weedy rice, which causes considerable loss in yield in the former. However we have not observed any crossover, despite growing *Oc* side by side with *Os* over several years. Attempts at hybridization between the two was also not successful (Islam et al., 2017; Maisha et al., 2023). We have observed that *Oc* desalinates the water, where it grows (**Supplementary Figure 1**). These observation are consistent with the fact that the wild halophyte uses Na^+^ uptake to the shoot to maintain osmotic adjustment (Ishikawa et al., 2022). This desalinization property of Oc likely helps the glycophytic rice to grow and perform better when co-habiting with the wild rice.

The work of Mishra and coworkers (2023) shows the ameliorative effect of halophytic tree plantation by decrease of alkalinity and ionic conductivity in soil as a result of improved soil organic carbon and microbial activity. A diverse range of endophytic bacteria was isolated from both wild rice and commercial rice under different conditions in the current work. Among them six different genera (Bacillus, Microbacterium, Achromobacter, Paraburkholderia, Aeromonas, Pseudomonas) were isolated from commercial rice and seven different genera (Bacillus, Aeromonas, Achromobacter, Microbacterium, Staphylococcus, Oerskovia, Cellulomonas) from wild rice from both control (unpaired) and paired conditions under 0mM and 100mM salt stress. Many of these bacteria were shown to have plant growth promoting activities (**Supplementary Table 1**). We have also shown how the Aeromonas and Achromobacter genera of endophytic microbes associated with the roots and rhizomes of *O. coarctata* transfer to the roots of the commercial cultivar in the pairing experiments (**Figure 3**). A previous study reported that Aeromonas species have potential as plant growth promoting endophytic bacteria, producing compounds such as indole acetic acid and cytokinins, and can improve tolerance to abiotic stresses such as salt (Meena et al., 2017). The Achromobacter genus has demonstrated substantial *in vitro* potential for ACCD (1-aminocyclopropane-1-carboxylate deaminase) activity, synthesis of indole compounds, and phosphate solubilization up to 100 mM NaCl concentration in the culture medium (Shahid et al., 2020). Bacillus strains have been shown to have a number of beneficial effects on rice plants, such as promoting growth, increasing nutrient uptake, and protecting against pathogens (Ali et al., 2021; Shultana et al., 2021). Pseudomonas strains have been found to be effective biocontrol agents against several rice pathogens, and some Pseudomonas strains are also known to produce plant growth-promoting compounds such as indole acetic acid, cytokinins and can fix nitrogen (Ali et al., 2021). The growth stimulation by the microorganisms can be a consequence of nitrogen-fixation or production of phytohormones and siderophores, biocontrol of phytopathogens, nutrient competition and inducing systemic acquired host resistance or immunity or by enhancing the availability of minerals nutrients (Bloemberg and Lugtenberg, 2001; Yang et al., 2009; Naher et al., 2018; Yadav, 2020). Besides pairing seems to induce the growth of roots and rhizomes of *O. coarctata* both with and without salt and can be considered as an increase in organic carbon in the environment (**Figures 3A, B**). We have also included a graph of how the co-growth of the halophyte with the commercial rice more effectively reduces the salinity of the surrounding environment (**Supplementary Figure 1**). Presence of plant growth promoting microbes and their use to improve growth of sensitive crops has also been shown by Etesami and Beattie (2018).

After convincingly observing growth promotion of the commercial rice cultivar in both the seedling and reproductive stages in the paired condition, we set out to find the differential expression of genes between the paired versus unpaired. Again the objective was to discover which genes were induced in the commercial cultivar as a result of the close proximity of the facultative halophytic wild rice, *O. coarctata*. In this study, a large number of DEGs were identified which provided clues on transcriptional regulation of gene response in the paired versus unpaired plants. Differential expression in the control paired group showed increased expression in photosynthesis, cellular biosynthesis and redox related genes. There was a significant differential upregulation of photosynthesis related genes in control paired e.g. chlorophyll a/b binding proteins which has been known to confer abiotic stress tolerance in Arabidopsis plants (Xu et al., 2011). In our study, the expression of this gene is 5 times higher in paired control in comparison to unpaired *O. sativa*. Another important DEG is NDR1/HIN1-like protein 1(LOC4351561), which has been shown to be 9 times higher in paired *O. sativa* compared to unpaired ones. The NHL (NDR1/HIN1-like) gene family includes specific genes in *Arabidopsis thaliana*, namely Harpin-induced gene 1 (HIN1) and Nonrace-specific disease resistance gene 1 (NDR1) (Century et al., 1997). HIN1 is activated by the presence of harpin protein and assumes an important role in plant defense responses, growth, development, and the ability to withstand abiotic stresses (Liu M. et al., 2020).

Salt stress treated paired *O. sativa* in comparison to unpaired ones showed increased expression of cell signaling and transport related genes. Most interestingly, we have found a significant number of DEGs involved in calcium signaling e.g. plasma membrane type calcium -transporting ATPase showed an increase of 4.6 fold (LOC_Os03g10640.1), calmodulin-binding protein 25-like (6 fold change) and endoplasmic reticulum calcium-transporting ATPase 4 (4.12 fold change) (LOC_Os05g02940.1). P2- type ATPases are believed to maintain low calcium cytosolic levels and are generally believed to have roles in abiotic stresses via calcium mediated signaling pathways. The expression of various P2- type ATPases was found to be upregulated under various abiotic stresses (Wang et al., 2022). Exclusively, our study found a gene called calcium-transporting ATPase 5, plasma membrane-type which is differentially upregulated only after salt treatment in paired *O. sativa*. Huda et al., 2013 demonstrated in transgenic tobacco lines that overexpression of this gene showed increased proline accumulation as well as ROS scavenging, thus promoting salt and drought tolerance (Haque et al., 2022). We did not find any significant expression of this gene in the unpaired groups for both control and salt conditions. The upregulation of peptidyl-prolyl cis-trans isomerase, PPIase (LOC_Os10g06630.1) in paired plants, even without stress treatment, may potentially be linked to the presence of *O. coarctata* which is already primed for salt tolerance. In our study, we detected 3 times higher expression of PPI in the paired salt vs control *O. sativa*. This PPIase belongs to the cyclophilin-like protein group and aids in stabilizing the cis-trans transition during protein folding, thus playing a role in enhancing tolerance to different forms of abiotic stress. (Kumari et al., 2009; Sekhar et al., 2010; Kaur et al., 2015)

We also found differentially expressed arabinogalactan proteins (AGPs) in paired *O. sativa* under salt stress. AGPs are known to play a role in cell expansion as cells need to readjust their size in response to salt stress. Lamport et al. (2006), noticed a significant increase in AGPs in tobacco cells exposed to salt stress. The rate of AGP release was found to be six times higher in salt-adapted cells, indicating a substantial increase in the diffusion rate of AGPs through a more porous pectic network (Mareri et al., 2019).

In control paired vs unpaired, a number of ABC transporter families (B, C and G) and their isoforms were found to be upregulated whereas some of them were downregulated after exposure to salt stress. One of them is ABC transporter B family member 4 isoform (Os01g0695625) which is downregulated under salt stress. ABCB and ABCG families are more frequently downregulated under drought and submergence stress (Nguyen et al., 2014). Plant transcription factors (TFs) exhibit numerous roles in abiotic and biotic stress responses. TF families like WRKY, bZIP, ERF, and MYB are vital in governing the expression of stress-related genes (DalCorso et al., 2010). In control paired *O. sativa*, WRKY, MYB, NAC, ERF are upregulated 3 fold higher in comparison to unpaired ones. Among them, the NAC transcription factor is 4.28 times higher in paired control plants. In Arabidopsis, transcription factor NAC, is a downstream TF of the auxin and ethylene signaling pathway. Overexpression of AtNAC2 led to lateral root development and can be induced by high salinity as well as abscisic acid (He et al., 2005). In addition, another transcription factor bHLH was upregulated 2.5 fold higher in paired *O. sativa* vs unpaired ones. Some bHLH TFs are found to be associated with salt tolerance by regulating the ion transporter Na^+^/H^+^ antiporter NHX1. In Arabidopsis, bHLH TF AtbHLH122 acts as an upstream regulator of AtNHX6, thus conferring salt tolerance (Krishnamurthy et al., 2019).

Overall, the KEGG pathway in control paired vs unpaired *O. sativa*, showed a higher number of biosynthesis related pathways. The metabolic pathway (osa01100), carbohydrate metabolism, sucrose and starch metabolism, etc. were activated and upregulated whereas in salt treated paired vs unpaired *O. sativa*, these pathways were downregulated except the upregulation of cellular signaling pathway e.g. MAPK (osa04016) and phosphatidylinositol signaling system (osa04070), etc. An example is the MAPK signaling cascade, which plays an important role in plant biotic and abiotic stress response by regulating some transcription factors (Pedley and Martin, 2005). Some WRKY TFs (osa: 9267508) have been shown to be positively or negatively regulated by MAPKs leading to plant immunity (Eulgem and Somssich, 2007). Our study showed that WRKY 22 like, WRKY 24, WRKY 30, WRKY 50, WRKY 70 are upregulated in control paired vs unpaired *O. sativa*. After exposure to salt, interestingly WRKY 22 like and 24 are found to be downregulated. During salt stress, the plant hormone auxin regulates numerous aspects of plant growth and development by facilitating the breakdown of Auxin/Indole-3-acetic acid (Aux/IAA) proteins. The expression of OsIAA4 and OsIAA11 has been shown to be induced by salt stress in rice, and they are thought to play a role in the plant’s response to this type of stress (Song and Xu, 2013; Chen et al., 2017). In our study upregulation of the auxin-responsive protein OsIAA18 gene was found in paired sativa plants under stress conditions. In addition, the auxin-responsive factor 2 (osa: 4325895) was upregulated in control conditions which was also validated by qRT-PCR.

Our validation of RNA sequencing (RNA-seq) data demonstrated consistent patterns of gene expression, which were confirmed on four randomly selected genes except JUNGBRUNNEN 1 (JuB) which belongs to the NAC transcription factor group. This exhibited an opposite expression pattern. NAC transcription factors are a large family of regulatory proteins in plants, known to play crucial roles in the transcriptional reprogramming associated with plant stress responses (Nuruzzaman et al., 2013). Thirumalaikumar et al. demonstrated that AtJUB1, also known as JUB1 (ANAC042) in *Arabidopsis thaliana*, plays a pivotal role as a key regulator of plant longevity and the intricate balance between growth and stress responses (Thirumalaikumar et al., 2018). During the validation process, we conducted separate experiments to collect RNA samples, which resulted in delays of sample collection compared to the RNA-seq data. It can be one a possible reason for the expression level of the JuB gene (LOC107277389) to show a contrasting trend compared to the other genes analyzed.

Overall it was observed that transporters, photosynthesis, and carbohydrate metabolism were generally upregulated in commercial *O. sativa* paired with the wild halophytic rice, *O. coarctata in* comparison with the unpaired even under 100mM salt stress. The differentially expressed genes involved in these metabolic pathways likely help the sensitive rice tackle salt stress better. Therefore, our mutualism experiment can be tried as an additional/alternative way of providing better growth and stress management of cultivable rice.

This study demonstrated that *O. coarctata* not only serves as a valuable resource of salt tolerant genes for its own survival but also plays a crucial role in enhancing salt stress defense in cohabitating sensitive plants. These beneficial effects are achieved through various interactions between *O. coarctata* and its neighboring plants, without any genetic modifications. We also showed that endophytic bacteria associated with *O. coarctata* may be involved in this beneficial effect of the wild halophyte on commercial rice.

## Conclusion

From the study, we can infer that mutualism between *O. coarctata* and cultivated rice (*O. sativa*) can significantly lower salt damage in rice seedlings and improve growth at the reproductive stage. However, appropriate cultivation strategies like devising suitable irrigation by *Oc*- conditioned water may need to be designed for the field, since *Oc* is basically a weedy plant and would need to be confined in separate plots. The specific *Oc* associated endophytes may also be used as biofertilizers to improve rice crop yields and establish a robust crop variety in regions of high salinity in the future.

## Data availability statement

The datasets presented in this study can be found in online repositories. The names of the repository/repositories and accession number(s) can be found in the article/**Supplementary Material**.

## Author contributions

AT and TA designed and performed the experiments. AT and IJ interpreted the RNA seq data, performed RT-qPCR and drafted the manuscript. AT compiled the physiological data, did the analyses and graphical representation of all data. DK and AT isolated and identified the endophytes. ZIS provided the overall concept and guided the planning, design and execution of the experiments, edited the manuscript and obtained the funds. All authors contributed to the article and approved the submitted version.
