# Supplementary material for: Paired growth of cultivated and halophytic wild rice under salt stress induces bacterial endophytes and gene expression responses
Source: Front Plant Sci. 2023 Sep 6;14:1244743. doi: 10.3389/fpls.2023.1244743 (PMC10516563; doi:10.3389/fpls.2023.1244743)
Supplement: Supplementary file 1 [file Table_1.docx]

**Supplementary File**

**Induction of genes and endophytic bacterial growth in commercial rice (*Oryza sativa*) during its paired growth with halophytic wild rice (*Oryza coarctata*) under salt stress**

Anika Tasnim^1^, Israt Jahan^1^, Tomalika Azim^1^, Dola Karmoker^1^, and Zeba I. Seraj^1*^

^1^Plant Biotechnology Laboratory, Department of Biochemistry and Molecular Biology, University of Dhaka, Dhaka, Bangladesh

***Corresponding author**: E-mail address: [zebai@du.ac.bd](mailto:zebai@du.ac.bd); Phone: +880 1711595576; ORCID: 0000-0002-1702-8574

**1 Supplementary Data**

**Supplementary File 1:** Quality control of clean raw reads of transcriptomics data.

**Supplementary File 2:** GO mapping of DEGs in both salt treatment and control conditions between paired and unpaired conditions of *O. Sativa*. GO mapping of DEGs in salt stress and control conditions, top ten up and down regulated genes in salinity treatment are shown in four separate worksheets of the supplementary excel file.

**Supplementary File 3:** KEGG mapping of DEGs in both salt treatment and control condition between paired and unpaired conditions of *O. Sativa*. The data in both control and salt condition are shown in two separate worksheets of the supplementary excel file.

**Supplementary File 4:** Phenotypic data collected from the *O. sativa* from different conditions. The data from both seedling and reproductive stages are shown in two separate worksheets of the supplementary excel file.

**2 Supplementary Table**

**Supplementary Table 1: Characterization of selected endophytic bacteria.**

| **Condition** | **Bacteria** | **1200mM salt tolerance test** | **N_2_ Fixation** | **N_2_+1200 mM salt stress** | **Phosphate solubilization** | **Phosphate solubilization+1200 mM salt stress** | **Zinc** | **Zinc+1200mM salt stress** | **Indole without salt** | **Indole in the presence of salt** |
| --- | --- | --- | --- | --- | --- | --- | --- | --- | --- | --- |
| **Coarctata_Rhizome_low salt stress** | PRhL1*(Aeromonas dhakensis)* | **+** | **+++** | **++** | **+** | **+** | **+++** | **_** | **++** | **++** |
| **Coarctata_Rhizome_Control** | PRhC2(*Cellulomonas hominis)* | **+** | **+++** | **+++** | **-** | **-** | **-** | **-** | **+** | **+** |
| **Coarctata_Root_Control** | PRC3(*Achromobacter xylosoxidans)* | **+** | **+** | **-** | **-** | **-** | **-** | **-** | **++** | **++** |
| **Coarctata_Rhizome_low salt stress** | PRhL2( *Bacillus altitudinis)* | **-** | **++** | **+++** | **+** | **+** | **++** | **-** | **+** | **-** |
| **Paired_Coarctata_Rizome_control** | PB6RhC1(*Bacillus stratosphericus* ) | **+** | **+++** | **+++** | **+** | **+** | **+** | **-** | **+** | **+** |
| **Paired_Coarctata_Rizome_control** | PB6RhC2(*Staphylococcus warneri*) | **+** | **++** | **++** | **+** | **+** | **-** | **-** | **+** | **+** |
| **Paired_Coarctata_Root_High salt stress** | PB6RH1(*Oerskovia paurometabola )* | **+** | **+** | **+** | **-** | **-** | **+** | **+** | **-** | **-** |

**Here the ‘+++’ indicates the highest activity, ‘++’ moderate activity and ‘+’ low activity and ‘-’ indicates negative results of activity.**

**3 Supplementary Figures**

**
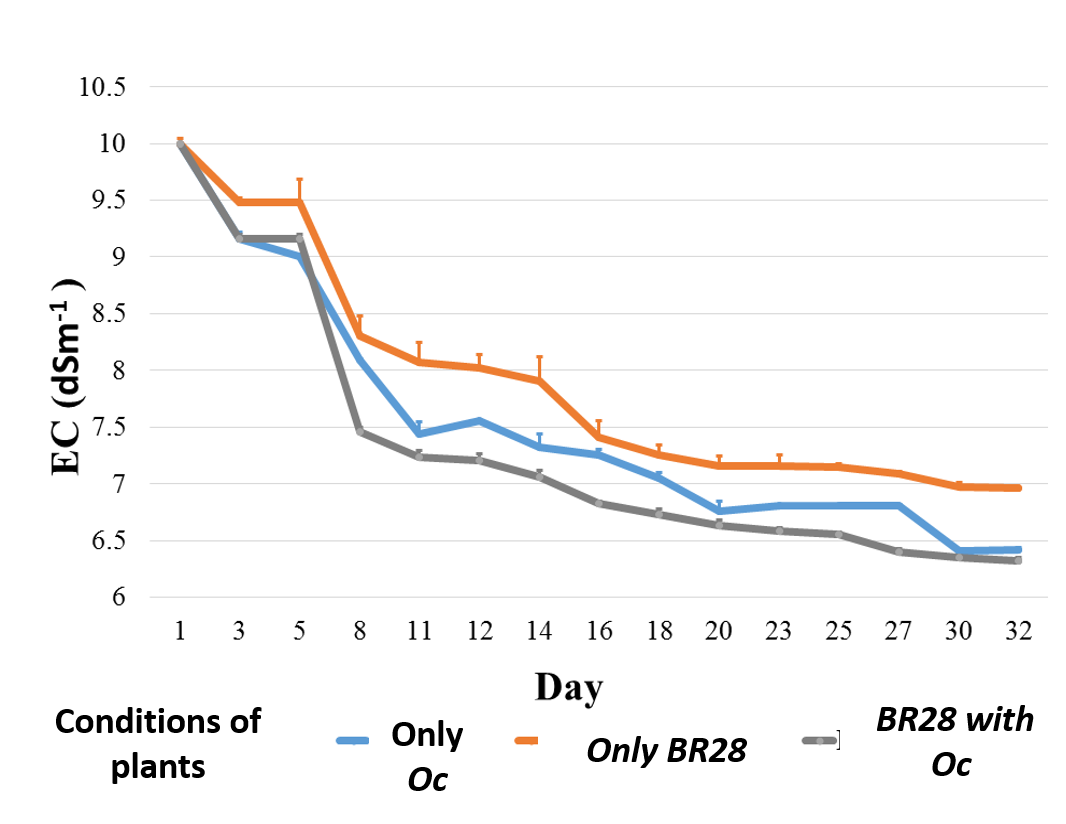
**

**Supplementary Fig 1: Desalinization activity measurement from mutualism experiment.**

**
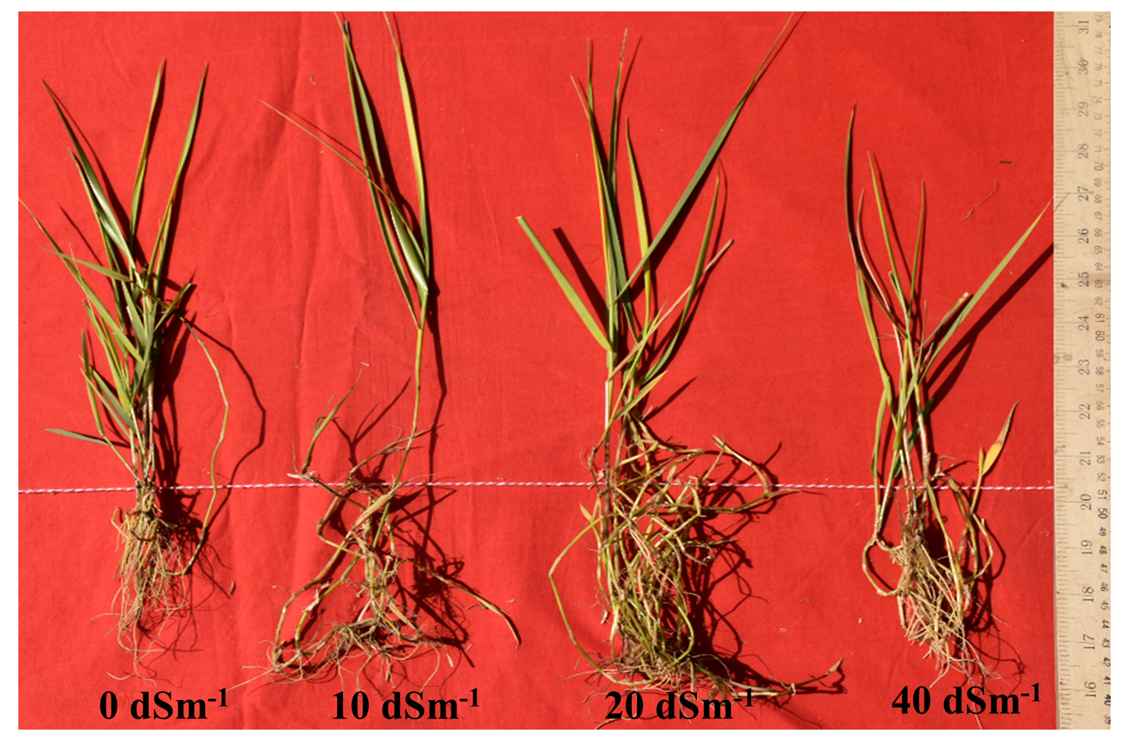
**

**Supplementary Fig 2: Observation of notable increase of rhizome in *O. coarctata* under high salinity level (200mM)**


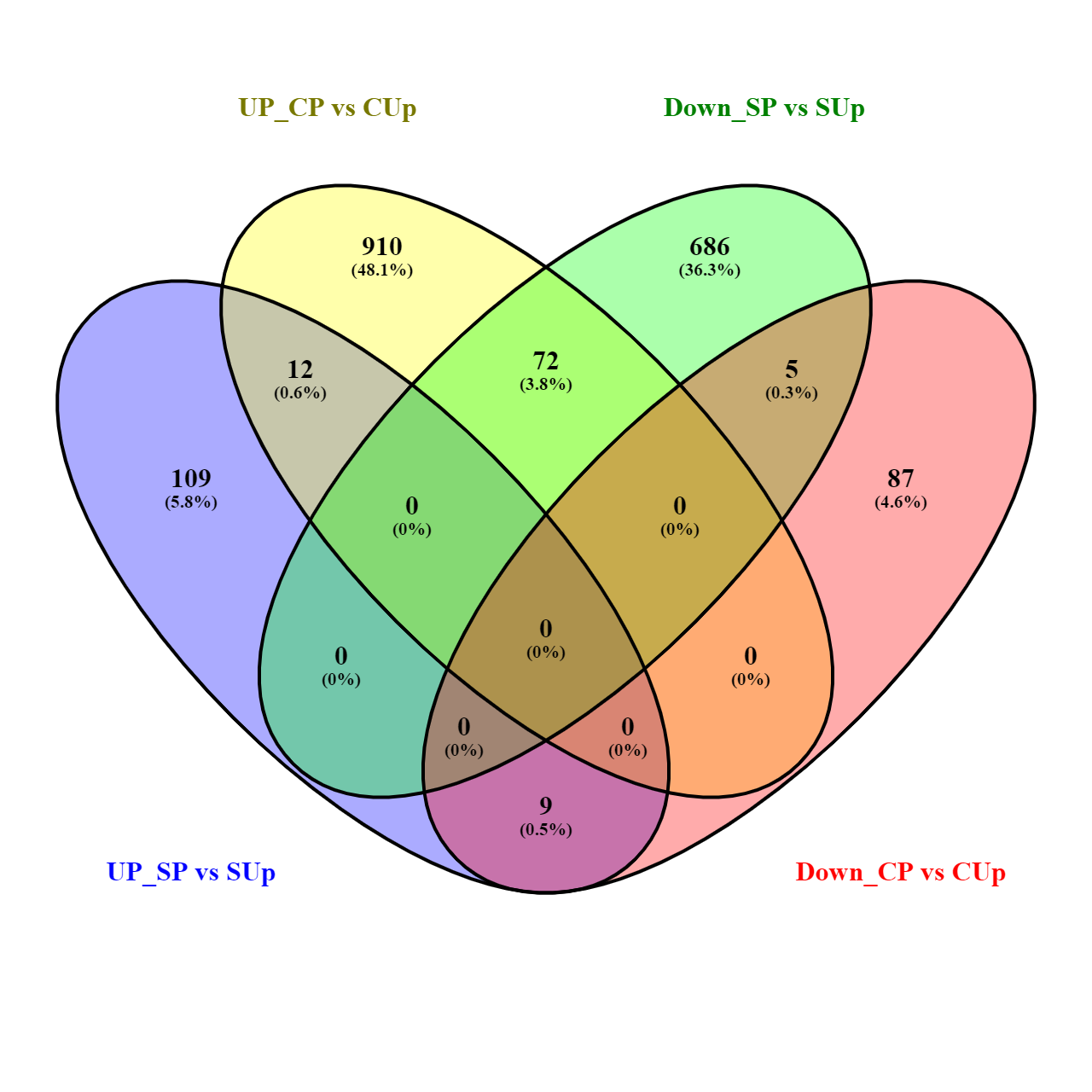


**Supplementary Fig 3: Venn diagram of Unique and Overlapping genes of differentially upregulated and downregulated in the case of *O. sativa*. Here, UP_CPvsCUp, Down_SP vs SUp, UP_SPvsSUp and Down_CPvsCUp indicate upregulated in control pair vs unpair, downregulated in salt pair vs unpair, Upregulated in salt pair vs unpair and downregulated in control pair vs Unpair respectively.**

**
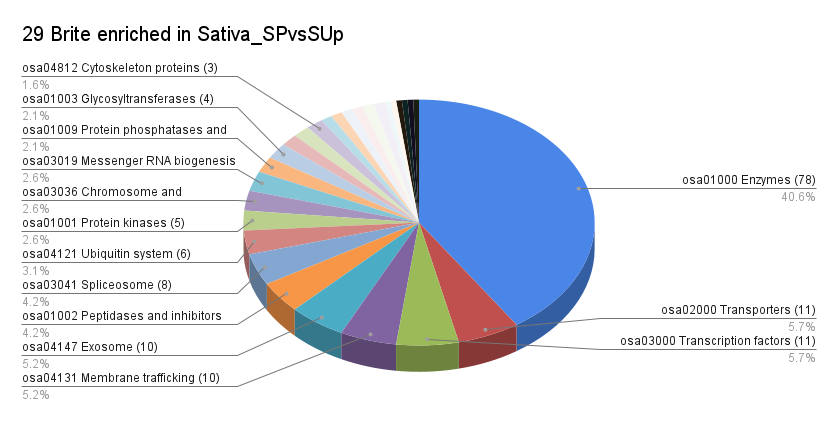
**

**Supplementary Fig 4: Brite enrichment analysis from KEGG pathway in case of Sativa salt pair vs unpair.**

**
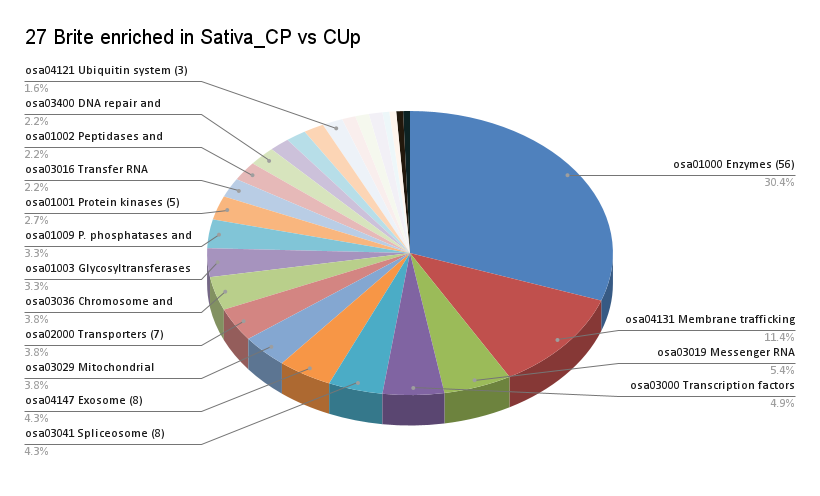
**

**Supplementary Fig 5: Brite enrichment analysis from KEGG pathway in case of Sativa control pair vs unpair.**

**
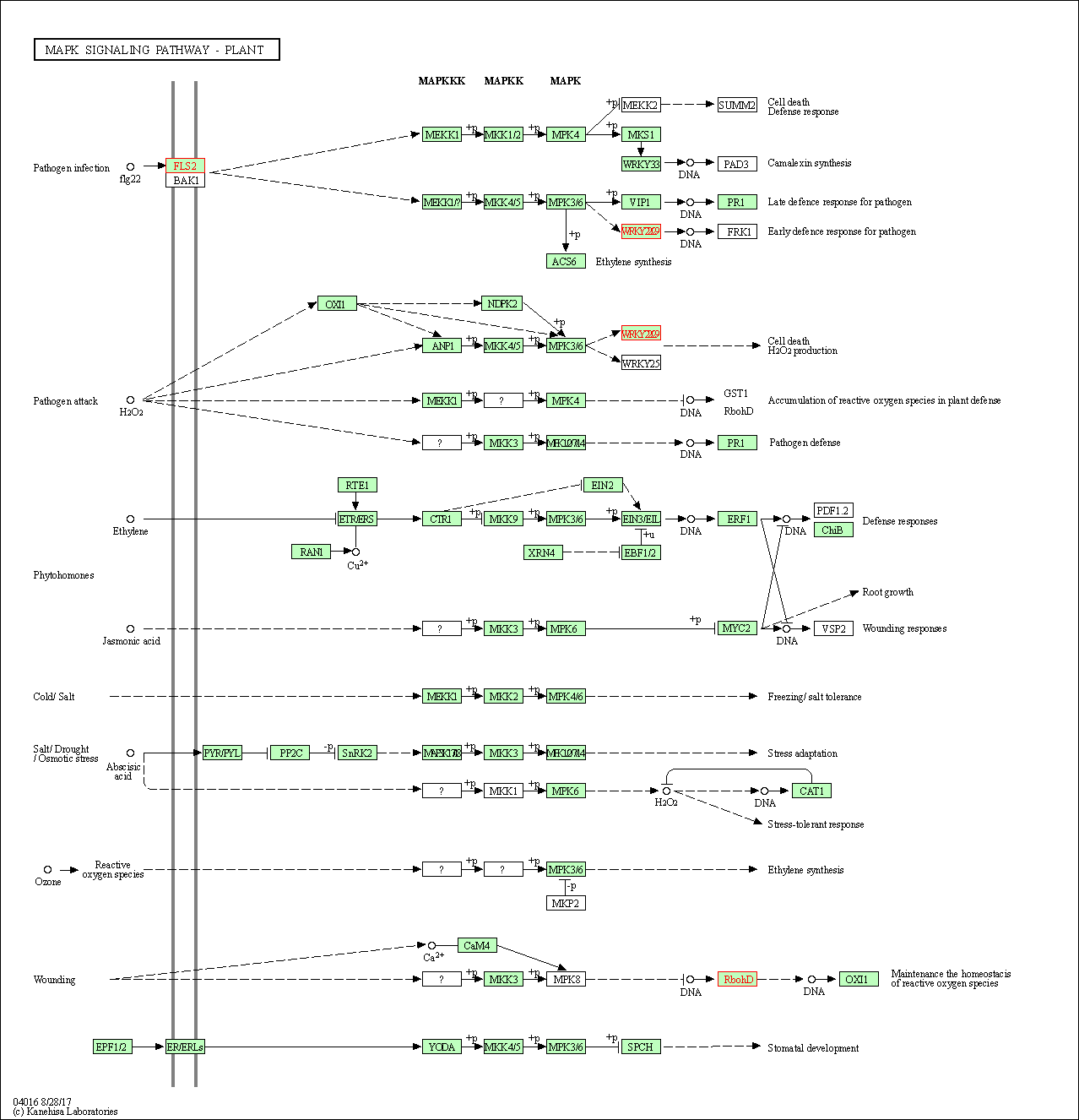
**

**Supplementary Fig 6: Mitogen-activated protein kinase (MAPK) signaling pathway in case of Sativa salt pair vs unpair.**

**
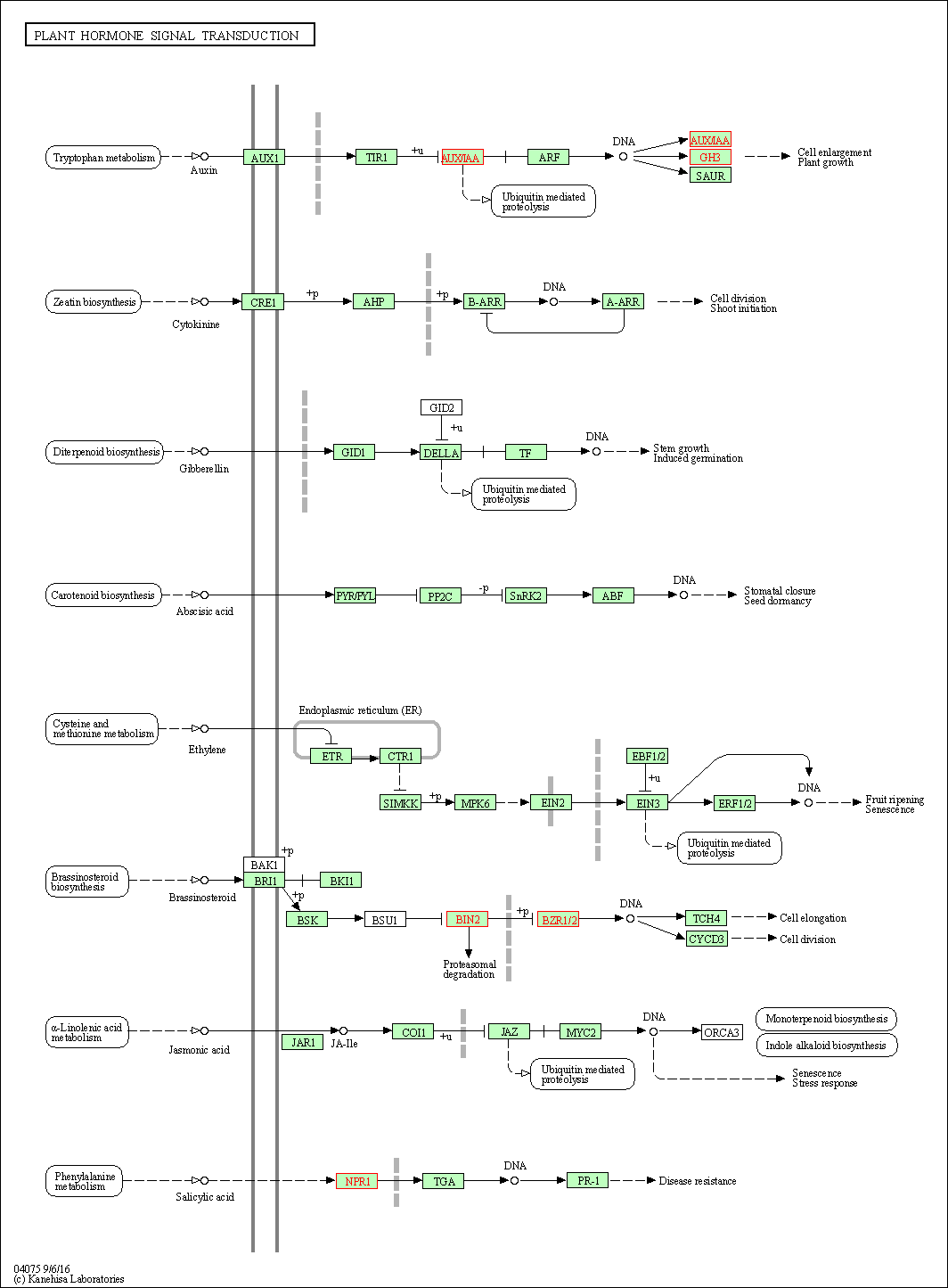
**

**Supplementary Fig 7: Plant hormone signal transduction pathway in case of Sativa salt pair vs unpair.**

**
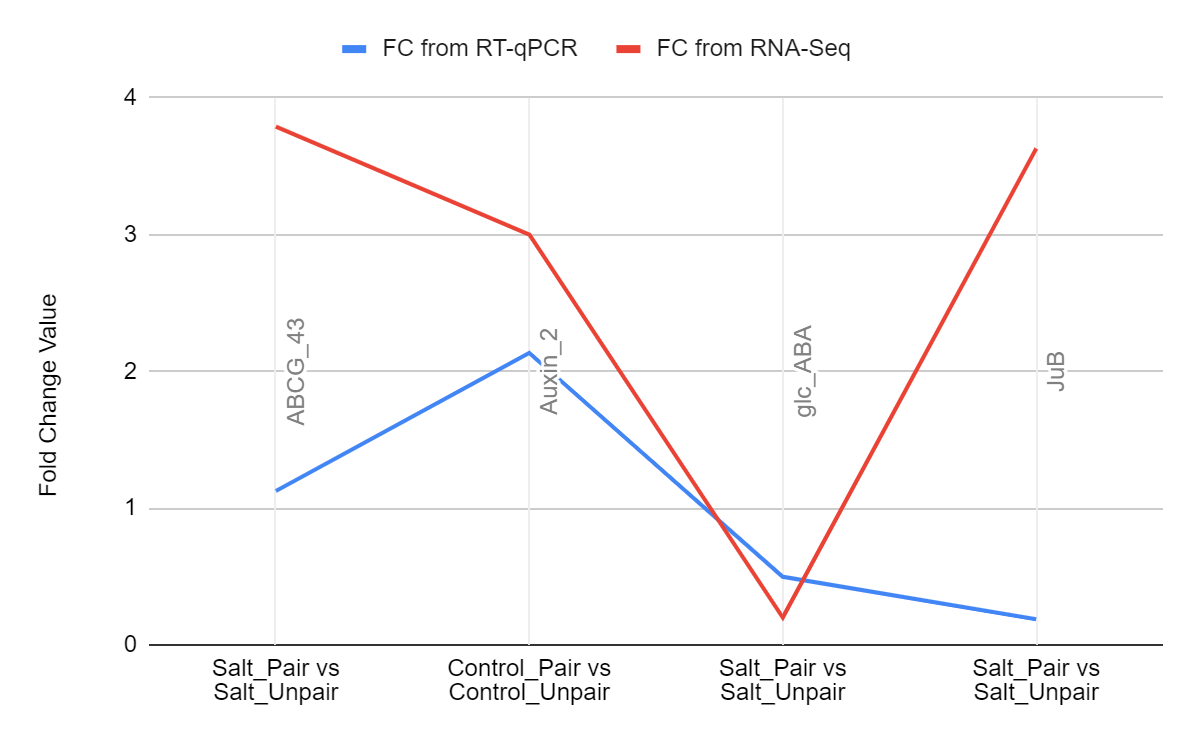
**

**Supplementary Fig 8: Representation of fold change value from RT-qPCR and RNA-seq of selected differentially expressed gene.**
